# Inanspruchnahme von Palliativversorgung am Lebensende in Deutschland: zeitlicher Verlauf (2016–2019) und regionale Variabilität

**DOI:** 10.1007/s00103-023-03683-7

**Published:** 2023-03-10

**Authors:** Bianka Ditscheid, Franziska Meissner, Cordula Gebel, Beata Hennig, Ursula Marschall, Winfried Meißner, Ulrich Wedding, Antje Freytag

**Affiliations:** 1 Institut für Allgemeinmedizin, Universitätsklinikum Jena, Bachstr. 18, 07743 Jena, Deutschland; 2 Abteilung Palliativmedizin der Klinik für Innere Medizin II, Universitätsklinikum Jena, Jena, Deutschland; 3 BARMER, Wuppertal, Deutschland

**Keywords:** Palliativversorgung, BQKPmV, SAPV, Hospiz, GKV-Routinedaten, Inanspruchnahme, Regionale Variation, Palliative (home) care, Specially qualified and coordinated palliative homecare, Specialized palliative homecare, Hospice, Claims data, Utilization, Regional variation

## Abstract

**Hintergrund:**

In Deutschland findet Palliativversorgung (PV) ambulant, stationär, allgemein und spezialisiert statt. Da bisher wenig bekannt ist über die zeitliche Entwicklung und regionale Unterschiede in den Versorgungsformen, war es Ziel der vorliegenden Studie, dies zu untersuchen.

**Methoden:**

Retrospektive Routinedatenstudie mit 417.405 in den Jahren 2016–2019 verstorbenen BARMER-Versicherten. Anhand mindestens einmalig abgerechneter Leistung im letzten Lebensjahr ermittelten wir die Inanspruchnahmeraten allgemeiner ambulanter Palliativversorgung (AAPV), besonders qualifizierter und koordinierter palliativmedizinischer Versorgung (BQKPmV), spezialisierter ambulanter Palliativversorgung (SAPV), stationärer Palliativ- und Hospizversorgung. Wir berechneten Zeittrends, regionale Unterschiede und kontrollierten für versorgungsbedarfsbezogene Patientenmerkmale und zugangsbezogene Wohnkreismerkmale.

**Ergebnisse:**

Von 2016 bis 2019 stieg die Inanspruchnahme von PV insgesamt von 33,8 % auf 36,2 %, SAPV von 13,3 % auf 16,0 % (max.: Rheinland-Pfalz), stationärer PV von 8,9 % auf 9,9 % (max.: Thüringen); AAPV sank von 25,8 % auf 23,9 % (max.: Brandenburg); BQKPmV kam 2019 auf 4,4 % (max.: Saarland); Hospiz blieb konstant bei 3,4 %. Die regionale Variabilität der Inanspruchnahmeraten nahm bei AAPV und stationärer PV von 2016 auf 2019 zu, bei SAPV und Hospiz ab, blieb insgesamt jedoch hoch. Die regionalen Unterschiede zeigten sich auch nach Adjustierung.

**Diskussion:**

Zunehmend mehr SAPV, weniger AAPV und hohe, nicht durch bedarfs‑/​zugangsbezogene Merkmale erklärbare regionale Variabilität sprechen dafür, dass sich der Einsatz palliativer Versorgungsformen weniger am Bedarf als an regional verfügbaren Versorgungskapazitäten orientiert. Angesichts demografiebedingt wachsenden PV-Bedarfs und abnehmender personeller Ressourcen ist diese Entwicklung kritisch zu sehen.

**Zusatzmaterial online:**

Zusätzliche Informationen sind in der Online-Version dieses Artikels (10.1007/s00103-023-03683-7) enthalten.

## Abstract (en)

**Background:**

In Germany, palliative care (PC) is provided on a homecare, inpatient, general, and specialized basis. Since little is currently known about the temporal course and regional differences in the forms of care, the present study was aimed to investigate this.

**Method:**

In a retrospective routine data study with 417,405 BARMER-insured persons who died between 2016 and 2019, we determined the utilization rates of primary PC (PPC), specially qualified and coordinated palliative homecare (PPC+), specialized palliative homecare (SPHC), inpatient PC, and hospice care on the basis of services billed at least once in the last year of life. We calculated time trends and regional variability and controlled for needs-related patient characteristics and access-related county of community characteristics.

**Results:**

From 2016 to 2019, total PC increased from 33.8 to 36.2%, SPHC from 13.3 to 16.0% (max: Rhineland-Palatinate), and inpatient PC from 8.9 to 9.9% (max: Thuringia). PPC decreased from 25.8 to 23.9% (min: Brandenburg) and PPC+ came in at 4.4% (max: Saarland) in 2019. Hospice care remained constant at 3.4%. Regional variability in utilization rates remained high, increased for PPC and inpatient PC from 2016 to 2019, and decreased for SPHC and hospice care. The regional differences were also evident after adjustment.

**Conclusion:**

Increasingly more SPHC, less PPC, and high regional variability, which cannot be explained by demand- or access-related characteristics, indicate that the use of PC forms is oriented less to demand than to regionally available care capacities. In view of the growing need for palliative care due to demographic factors and decreasing personnel resources, this development must be viewed critically.

## Einleitung

Nach wie vor mangelt es der öffentlichen Berichterstattung zur Palliativversorgung (PV) an einer vollständigen Erfassung palliativer Versorgungsleistungen. Entscheidungen über die Weiterentwicklung der PV in Deutschland benötigen als rationale Entscheidungsgrundlage Daten darüber, wie viele und welche Menschen in einer Region am Lebensende in welcher Form palliativ versorgt wurden und wie sich diese Zahlen über die Zeit entwickelt haben.

Die letzte deutschlandweite Bestandsaufnahme mit räumlicher Differenzierung nach den Regionen kassenärztlicher Vereinigungen (KVen) zur Inanspruchnahme palliativer Versorgungsformen am Lebensende berichtete über das Jahr 2016 [1]. Die zwischen den KVen sehr unterschiedlichen Inanspruchnahmeraten warfen u. a. die Frage auf, inwieweit diese durch regional unterschiedlich verteilten Versorgungsbedarf der Patienten^1^ begründet sind. Mit dem vorliegenden Update schreiben wir die Zeitreihe bis 2019 fort. Außerdem prüfen wir anhand der uns vorliegenden empirischen Daten, ob die Variation der PV-Inanspruchnahmeraten zwischen den KVen durch regional unterschiedlich verteilte, auf Versorgungsbedarf und -zugang bezogene Patienten- bzw. Wohnkreismerkmale gegründet ist.

## Methoden

Wir führten eine retrospektive Kohortenstudie mit Routinedaten der BARMER über Versicherte mit Sterbedatum in den Jahren 2016 bis 2019 durch: Anhand einer mindestens einmalig abgerechneten Leistung im letzten Lebensjahr ermittelten wir die Inanspruchnahmeraten allgemeiner ambulanter Palliativversorgung (AAPV), besonders qualifizierter und koordinierter palliativmedizinischer Versorgung (BQKPmV), spezialisierter ambulanter Palliativversorgung (SAPV) sowie stationärer Palliativ- und Hospizversorgung auf Bundes- und KV-Ebene. Zusätzlich berechneten wir weitere Kennzahlen im Zusammenhang mit der PV: u. a. SAPV-Verordnungen aus dem Krankenhaus, palliative häusliche Krankenpflege, Tageshospize, PV-Leistungen im Rahmen der Vereinbarung über die qualifizierte ambulante Versorgung krebskranker Patienten „Onkologie-Vereinbarung“ [2], ambulante geriatrische Leistungen. Wir bestimmten die Trends für die Entwicklung der Inanspruchnahme im Zeitraum 2016–2019 und die Abweichungen der KVen vom Mittel der KVen sowie den Variationskoeffizienten (VK) zwischen den KVen als Maß für die Variabilität. Wir adjustierten die Inanspruchnahmeraten um versorgungsbedarfsbezogene, in Routinedaten der gesetzlichen Krankenversicherung (GKV-Routinedaten) verfügbare Patientenmerkmale (Alter, Geschlecht, pflegerische Versorgung, Gesamtmorbidität, potenziell ursächliche bzw. Versorgungsbedarf begründende chronische Grunderkrankungen) sowie um versorgungszugangsbezogene Wohnkreismerkmale (sozioökonomischer Deprivationsgrad (GISD) und Ländlichkeitsgrad^2^). Alle Analysen beruhen auf alters- und geschlechtsstandardisierten Daten. Die Ergebnisdarstellung erfolgt nach international anerkannten Kriterien für Beobachtungsstudien (STROBE; [3]). Details zur Methodik sind im Onlinematerial ausführlich beschrieben.

## Ergebnisse

### Studienpopulation

Insgesamt konnten die Daten von 417.405 BARMER-Versicherten, die 2016 bis 2019 verstarben, eingeschlossen werden. Im Onlinematerial finden sich Übersichten über die Zahl der eingeschlossenen Verstorbenen je Jahr und KV (Tab. B-1), die Zusammensetzung der Studienpopulation nach Alter, Geschlecht, Pflegebedürftigkeit, Morbidität, chronischen Grunderkrankungen (Tab. B-2) sowie der Anteil der eingeschlossenen Verstorbenen an den in Deutschland insgesamt dokumentierten Sterbefällen nach Bundesländern (Tab. B-3). Die Ausprägung der Adjustierungsparameter bei den Verstorbenen der 17 KVen zeigen Tab. B‑4 und B‑5 (Patientenmerkmale), die Wohnkreismerkmale je KV sind in Tab. B‑6 zu finden (siehe Onlinematerial).

### Inanspruchnahme von Palliativversorgung

#### Entwicklungen auf Bundesebene

**Figure 1:**
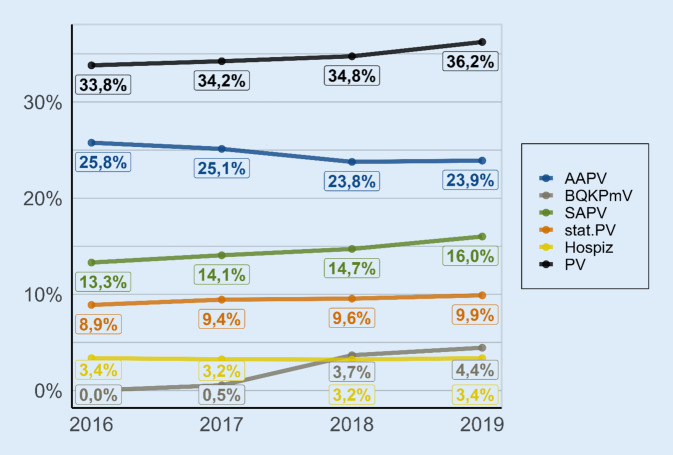

Die Inanspruchnahme von PV insgesamt (Abb. 1; Tab. B-7) nahm im Verlauf von 2016 bis 2019 signifikant zu, von 33,8 % auf 36,2 % (OR 1,04; *p* < 0,001; Tab. 1). Der Anteil der Versicherten (VS), die im letzten Lebensjahr keine PV erhielten, nahm somit von 66,2 % (2016) auf 63,8 % (2019) ab.

**Table 1:** 

	AAPV				SAPV				Stat. PV				Hospiz				PV			
**Mittlerer Zeittrend**	**0,95**	***			**1,08**	***			**1,03**	***			**1,01**	**ns**			**1,04**	***		
	**Zeittrendabweichung**		**KV-spezifischer Zeittrend**		**Zeittrendabweichung**		**KV-spezifischer Zeittrend**		**Zeittrendabweichung**		**KV-spezifischer Zeittrend**		**Zeittrendabweichung**		**KV-spezifischer Zeittrend**		**Zeittrendabweichung**		**KV-spezifischer Zeittrend**	
Baden-Württemberg	1,00	ns	–		1,03	+	–		0,97	ns	–		0,99	ns	–		1,00	ns	–	
Bayern	1,04	***	0,98	*	0,98	ns	–		1,02	ns	–		0,90	**	0,91	**	1,00	ns	–	
Berlin	1,01	ns	–		0,95	**	1,02	ns	1,05	*	1,08	***	0,96	ns	–		1,00	ns	–	
Brandenburg	0,92	***	0,87	***	0,98	ns	–		1,01	ns	–		0,97	ns	–		1,00	ns	–	
Bremen	1,05	ns	–		1,04	ns	–		0,87	ns	–		1,01	ns	–		1,02	ns	–	
Hamburg	0,99	ns	–		0,98	ns	–		1,00	ns	–		0,97	ns	–		0,98	ns	–	
Hessen	0,97	**	0,92	***	0,98	ns	–		0,99	ns	–		1,03	ns	–		0,98	+	–	
Mecklenburg-Vorpommern	0,98	ns	–		0,97	ns	–		0,93	**	0,95	+	0,95	ns	–		0,98	ns	–	
Niedersachsen	0,98	+	–		0,98	+	–		0,99	ns	–		0,95	+	–		0,96	***	1,00	ns
Nordrhein	1,03	**	0,98	**	1,04	**	1,12	***	1,02	ns	–		0,98	ns	–		1,00	ns	–	
Rheinland-Pfalz	1,02	ns	–		1,10	***	1,19	***	0,99	ns	–		1,01	ns	–		1,01	ns	–	
Saarland	0,93	*	0,88	***	0,99	ns	–		1,05	ns	–		1,02	ns	–		1,02	ns	–	
Sachsen	1,04	**	0,99	ns	1,00	ns	–		1,04	+	–		1,05	ns	–		1,02	+	–	
Sachsen-Anhalt	0,93	**	0,89	***	0,96	+	–		0,97	ns	–		1,07	ns	–		0,99	ns	–	
Schleswig-Holstein	1,00	ns	–		1,00	ns	–		1,08	**	1,11	***	1,05	ns	–		1,00	ns	–	
Thüringen	1,02	ns	–		1,04	ns	–		1,06	*	1,08	**	1,08	+	–		1,03	ns	–	
Westfalen-Lippe	1,10	***	1,04	***	0,99	ns	–		0,99	ns	–		1,01	ns	–		1,02	+	–	

Dargestellt sind Odds Ratios (*OR*) des mittleren Zeittrends und der Abweichung der 17 KVen vom mittleren Zeittrend mit ihrer jeweiligen Signifikanz. Für KVen, deren Zeittrend signifikant (mit p < 0,05) vom mittleren Zeittrend abweicht, ist zusätzlich auch der KV-spezifische Zeittrend ausgewiesen

*AAPV* allgemeine ambulante Palliativversorgung, *KV* Kassenärztliche Vereinigung, *ns* nicht signifikant, *PV* Palliativversorgung, *SAPV* spezialisierte ambulante Palliativversorgung, *stat. PV* stationäre Palliativversorgung

***p < 0,001, **p < 0,01, *p < 0,05, ^+^*p* < 0,10

Während die im 4. Quartal 2017 neu eingeführte BQKPmV innerhalb von 2 Jahren auf einen Anteil von 4,4 % der VS im Jahr 2019 anwuchs, ging die Inanspruchnahme von AAPV zurück, von 25,8 % im Jahr 2016 auf 23,9 % im Jahr 2019 (OR 0,95; *p* < 0,001). Davon entfielen bis 2019 nahezu konstant 2,1 Prozentpunkte auf PV gemäß „Onkologie-Vereinbarung“ (Tab. B-8). Dies zeigt, dass die Abnahme bei der AAPV ausschließlich auf hausärztlich abgerechnete AAPV-Leistungen zurückgeht.

Tab. B‑9 im Onlinematerial zeigt die Inanspruchnahme von palliativen häuslichen Krankenpflegeleistungen (HKPpall), einschließlich der „Symptomkontrolle bei palliativen Patienten“, also pflegerische Leistungen, die im Allgemeinen der AAPV zugeordnet werden [4]. Deren Inanspruchnahmerate lag 2019 bundesdurchschnittlich bei nur 0,7 %. Werden sämtliche Leistungen der häuslichen Krankenpflege (HKP) nach § 37 Fünftes Buch Sozialgesetzbuch (SGB V) ab der ersten ambulanten Palliativleistung (HKP ab ambulanter PV) betrachtet, so gelangt man zu einer Rate von 5,0 %. Ambulante Pflege gemäß Elftem Buch Sozialgesetzbuch (SGB XI) erhielten 56,5 % der VS.

Die Inanspruchnahme von SAPV nahm zu, von 13,3 % im Jahr 2016 auf 16,0 % im Jahr 2019 (OR 1,08; *p* < 0,001; Abb. 1; Tab. B‑7 und Tab. 1). Der Anteil im Krankenhaus erstverordneter SAPV (SAPV-VO aus Krankenhaus) lag 2019 mit 23,8 % bei etwa einem Viertel der insgesamt in Anspruch genommenen SAPV vs. 24,1 % im Jahr 2016 (Tab. B-9), d. h., der Anteil der haus- oder fachärztlich verordneten SAPV nahm zu.^3^ Der Anteil der VS, die als ambulante palliativmedizinische Versorgung ausschließlich AAPV oder BQKPmV und nicht SAPV erhielten („maxAAPV oder maxBQKPmV“), betrug 2019 16,6 % (Tab. B-8). Dies war kaum mehr als der Anteil an VS, die (ausschließlich bzw. auch) SAPV erhielten. Bundesdurchschnittlich kam SAPV bei 6,3 % der VS ohne (gleichzeitige, vor- oder nachgelagerte) AAPV bzw. BQKPmV zum Einsatz (Tab. B-9).

Auch für die Inanspruchnahme stationärer PV zeigte sich eine Zunahme von 8,9 % im Jahr 2016 auf 9,9 % im Jahr 2019 (OR 1,03; *p* < 0,001), während die Inanspruchnahmerate von Hospizen gleich blieb mit 3,4 % im Jahr 2016 wie im Jahr 2019 (OR 1,01; ns). 2019 wie 2016 erfolgte gut ein Drittel aller stationären PV, ohne dass zuvor oder nachher ambulante PV zum Einsatz kam. Die in Entwicklung befindlichen neuen Versorgungsangebote wie Tageshospize wurden im betrachteten Zeitraum insgesamt nur vereinzelt (von *n* = 70 VS) in Anspruch genommen (Tab. B-9).

#### Entwicklungen auf Ebene der kassenärztlichen Vereinigungen (KVen)

##### Palliativversorgung (PV) gesamt.

**Figure 2:**
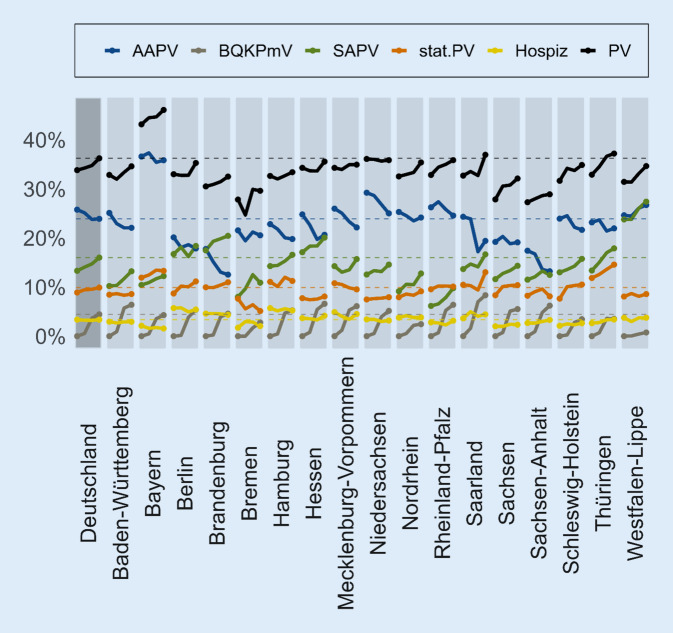

Einen grafischen Überblick über die Entwicklung der Inanspruchnahmeraten in den KVen von 2016 bis 2019 gibt Abb. 2, über den Vergleich der KVen im Jahr 2019 Abb. 3. Die deskriptiven Inanspruchnahmeraten der Jahre 2019 und 2016 finden sich in Tab. B‑7. Insgesamt verzeichnete Bayern 2019 wie schon 2016 die höchste Inanspruchnahmerate an PV mit 46,1 % der VS. Die geringste Rate entfiel wie schon 2016 auf Sachsen-Anhalt mit 28,9 %.

**Figure 3:**
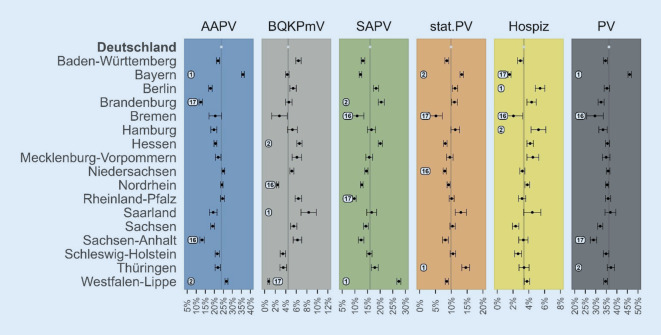

KV-spezifische Abweichungen vom mittleren Zeittrend über die betrachtete palliative Versorgungsform finden sich in Tab. 1. Dort sind außerdem die KV-spezifischen Zeittrends für die KVen angegeben, die sich signifikant vom Bundestrend unterscheiden. Danach verzeichnet nur die KV Niedersachsen einen vom Bundestrend abweichenden Zeittrend der Inanspruchnahme von PV; dieser ist nicht signifikant, also anders als der Bundestrend nicht zunehmend im Zeitverlauf.

##### Allgemeine ambulante Palliativversorgung (AAPV).

Am höchsten war der Anteil der VS mit AAPV 2019 in Bayern mit 35,8 %, am geringsten in Brandenburg mit 12,5 % (Abb. 2; Tab. B-7). Auch 2016 verzeichnete Bayern mit 36,6 % die höchste Inanspruchnahmerate, Sachsen-Anhalt mit 17,4 % die niedrigste und Brandenburg die zweitniedrigste mit 17,8 %. Dabei war der Abwärtstrend in Bayern geringer, der in Brandenburg stärker als der mittlere ebenfalls abnehmende Zeittrend des Mittels der KVen (Tab. 1). Fast ebenso stark rückläufig wie in Brandenburg war die AAPV im Saarland und in Sachsen-Anhalt. Die einzige KV, die bei der AAPV einen der mittleren zeitlichen Entwicklung gegenläufigen Trend aufweist, war Westfalen-Lippe: Dort stieg der Anteil der VS mit AAPV von 24,6 % (2016) auf 26,7 % (2019; OR 1,04; *p* < 0,001).

Tab. B‑8 zeigt darüber hinaus: Leistungen gemäß „Onkologie-Vereinbarung“ als Teil der als AAPV eingeschlossenen Leistungen wurden in den KVen ebenfalls sehr unterschiedlich häufig abgerechnet (2019: von 0,4 % der VS im Saarland bis 4,7 % in Berlin).

Palliative HKP-Leistungen (HKPpall) wurden 2019 fast ausschließlich in Westfalen-Lippe (3,7 %) und Nordrhein (1,9 %) abgerechnet (Tab. B-9). Die höchste Inanspruchnahme von HKP ab erster ambulanter PV verzeichnete hingegen Mecklenburg-Vorpommern mit 8,3 %, während diese Rate in Berlin und Hamburg jeweils nur bei 2,6 % lag. Die Inanspruchnahme ambulanter Pflege gemäß SGB XI im letzten Lebensjahr schwankte lediglich zwischen 52,4 % in Berlin und 59,7 % in Bremen.

##### Besonders qualifizierte und koordinierte palliativmedizinische Versorgung (BQKPmV).

Die höchste Inanspruchnahmerate für die im 4. Quartal 2017 neu eingeführte BQKPmV von 8,3 % wurde 2019 im Saarland erzielt, mit etwas Abstand gefolgt von Hessen mit 6,6 % (Abb. 2; Tab. B-7). In Westfalen-Lippe spielt die BQKPmV mit 0,8 % (2019) praktisch keine Rolle. Gering war die Inanspruchnahme von BQKPmV ebenfalls in Nordrhein mit 2,4 % der VS. In allen KVen nahm die BQKPmV-Inanspruchnahme über die Zeit zu.^4^

Betrachtet man BQKPmV und AAPV gemeinsam, d. h. die Inanspruchnahme von „AAPV oder BQKPmV“ (Tab. B-8), zeigt sich 2019 eine der AAPV-Inanspruchnahme ähnliche regionale Verteilung (höchste Rate in Bayern, niedrigste Rate in Brandenburg), mit einer Ausnahme: Das Saarland rutscht in dieser gemeinsamen Betrachtung um eine merkliche Anzahl von 5 Rängen nach „oben“ (von 13 auf 8).

Maximal-AAPV oder BQKPmV (und nicht SAPV) als ambulante PV erhielten die meisten VS in Bayern mit 28,6 % und die wenigsten in Westfalen-Lippe mit 4,1 % sowie in Brandenburg mit 7,7 % (Tab. B-8). Während dies in Brandenburg auf die niedrigen AAPV-/BQKPmV-Raten insgesamt zurückzuführen ist, werden AAPV und SAPV in Westfalen-Lippe integriert angeboten. Anstelle von SAPV-Teams übernehmen dort palliativmedizinische Konsiliardienste (PKD) die (spezialisierte) Palliativversorgung.

##### Spezialisierte ambulante Palliativversorgung (SAPV).

Die höchste Inanspruchnahme von SAPV (PKD-Versorgung) fand sich im Jahr 2019 wie schon 2016 in Westfalen-Lippe mit 27,4 % der VS. Dahinter folgte (wie auch schon 2016) Brandenburg mit 20,4 %. Die geringste Rate fand sich nach wie vor in Rheinland-Pfalz mit 9,8 %, jedoch mit der größten Zunahme 2016–2019 (OR 1,19; *p* < 0,001) und damit der größten Abweichung nach oben vom positiven Bundestrend. Ebenfalls überdurchschnittlich stieg die SAPV in Nordrhein an (OR 1,12; *p* < 0,001). Eine Verringerung der SAPV-Inanspruchnahme war in keiner KV zu verzeichnen (Tab. 1 und B-7).

SAPV kam gänzlich ohne AAPV oder BQKPmV am häufigsten bei 12,7 % der VS in Brandenburg und am seltensten bei 3,1 % der VS in Bayern zum Einsatz (Tab. B-9).

##### Stationäre Palliativversorgung (stat. PV).

Der höchste Anteil an stationärer PV war 2019 in Thüringen zu finden mit 14,6 % der VS mit einem über dem Bundestrend liegenden Wachstum im Zeitraum 2016–2019 (Tab. 1 und Tab. B-7). Die geringste Rate wiesen Bremen mit 5,1 % und Niedersachsen mit 7,9 % auf (Tab. B-7). Die stationäre PV nahm im Mittel aller KVen zu. Nur in Mecklenburg-Vorpommern fiel der Zeittrend signifikant schwächer aus und zeigte deskriptiv sogar eine abnehmende Tendenz (OR 0,95; ns). Der höchste Anstieg war in Schleswig-Holstein zu verzeichnen (OR 1,11; *p* < 0,001; Tab. 1). Teilweise kommt stationäre auch ohne (vorherige oder nachgelagerte) ambulante PV zum Einsatz. Dies war 2019 am häufigsten bei 5,2 % der VS in Bayern und am seltensten bei 1,9 % der VS in Bremen der Fall, gefolgt von Niedersachsen mit 2,6 % (Tab. B-9).

##### Hospiz.

Die Inanspruchnahme von stationären Hospizen blieb 2016–2019 sowohl im Bundesschnitt als auch in den KVen in etwa gleich (mit Ausnahme von Bayern, wo sie abnahm [OR 0,91; *p* < 0,01; Tab. 1]). Ins Auge fallen im Jahr 2019 (Tab. B-7) die hohen Inanspruchnahmeraten in Berlin (5,4 %) und Hamburg (5,2 %) und die geringe in Bayern (1,6 %). Von den VS mit Hospizinanspruchnahme erhielten 2019 im Bundesdurchschnitt 68,7 % auch SAPV, davon in einigen KVen (nahezu) alle (Berlin 98,6 %, Bremen 100 %).

##### Variabilität zwischen KVen.

Die Variabilität der PV-Inanspruchnahme zwischen den KVen im Jahr 2019 und ihre Veränderung von 2016 auf 2019 zeigt Tab. 2. Gemessen am Variationskoeffizienten (VK) war die Variabilität bei der BQKPmV mit 38,0 % am höchsten, gefolgt von der Hospizinanspruchnahme mit 30,3 %. In den übrigen palliativen Versorgungsformen, d. h. stationäre PV, AAPV und SAPV, war sie in etwa gleich hoch (VK 23,1 %, 24,3 % und 26,9 %) und auf die PV insgesamt bezogen am geringsten (VK 10,5 %). Für die AAPV und die stationäre PV hat sich die Variabilität von 2016 auf 2019 erhöht und sich somit die Unterschiedlichkeit zwischen den KVen vergrößert. Bei SAPV und Hospiz nahm die Variabilität ab, was andeutet, dass sich hier die KVen einander annähern.

**Table 2:** 

Versorgungsform	Mittel aller VS (%)	Mittel der KVen (%)	VK (%)	Höchste Rate (%)	Niedrigste Rate (%)	KV mit höchster Rate	KV mit niedrigster Rate	VK (Veränderung)	Höchste Rate (Veränderung)	Niedrigste Rate (Veränderung)
AAPV	23,9	21,6	24,3	35,8	12,5	Bayern	Brandenburg	+5,4	−0,8	−4,9
BQKPmV	4,4	4,9	38,0	8,3	0,8	Saarland	Westfalen-Lippe	–	+8,3	+0,8
SAPV	16,0	15,8	26,9	27,4	9,8	Westfalen-Lippe	Rheinland-Pfalz	−4,7	+3,6	+3,6
Stat. PV	9,9	10,0	23,1	14,6	5,1	Thüringen	Bremen	+6,2	+2,6	−2,4
Hospiz	3,4	3,5	30,3	5,4	1,6	Berlin	Bayern	−5,7	−0,3	−0,2
PV	36,2	34,9	10,5	46,1	28,9	Bayern	Sachsen-Anhalt	−0,6	+2,9	+1,6

Veränderung als Differenz der absoluten Werte von 2019–2016

*AAPV* allgemeine ambulante Palliativversorgung, *BQKPmV* besonders qualifizierte und koordinierte palliativmedizinische Versorgung, *KV* Kassenärztliche Vereinigung, *PV* Palliativversorgung, *SAPV* spezialisierte ambulante Palliativversorgung, *stat. PV* stationäre Palliativversorgung, *VK* Variationskoeffizient (Standardabweichung geteilt durch Mittelwert), *VS* Versicherte

#### Bedarfs- und zugangsadjustierte Inanspruchnahmeraten

In einem nächsten Schritt wurde geprüft, ob die im vorherigen Abschnitt berichteten Ergebnisse Bestand haben, auch wenn man für den Zusammenhang der regional variierenden versorgungsbedarfsbezogenen Patientenmerkmale und versorgungszugangsbezogenen Wohnkreismerkmale (vgl. Onlinematerial Abschnitt A.6 sowie Tab. B‑4 bis B-6) kontrolliert. Tab. B-10 zeigt, dass fast alle gewählten Adjustierungsparameter mit der Inanspruchnahme von PV insgesamt zusammenhängen. Die detaillierte Analyse und Interpretation dieser Zusammenhänge – bivariat wie multipel und differenziert nach den PV-Formen – ist weiteren Publikationen vorbehalten, wie zunächst [5, 6].

**Figure 4:**
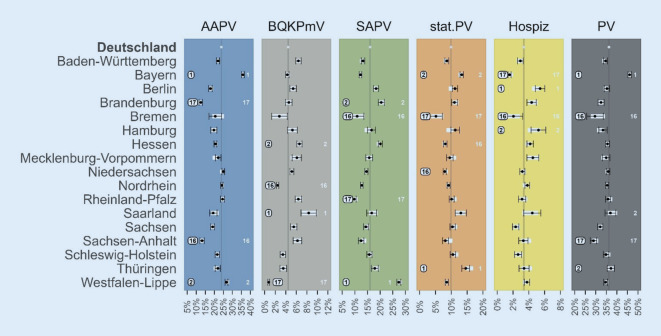

Es zeigt sich, dass sich die im vorherigen Abschnitt berichtete Rangfolge der KVen hinsichtlich der Inanspruchnahmeraten im Jahr 2019 kaum ändert, wenn man für versorgungsbedarfsbezogene Patientenmerkmale und versorgungszugangsbezogene Wohnkreismerkmale kontrolliert (Abb. 4). Lediglich bei der stationären PV weist dann nicht Niedersachsen, sondern Hessen die zweitgeringste Inanspruchnahme auf. Bei der PV insgesamt weist weiterhin Bayern die höchste, aber nicht mehr Thüringen, sondern das Saarland die zweithöchste Inanspruchnahme auf. Regionale Unterschiede in der Inanspruchnahme der jeweiligen PV-Formen lassen sich demnach nicht einfach durch die heterogen verteilten (gemessenen) versorgungsbedarfsbezogenen und versorgungszugangsbezogenen Merkmale erklären.

Auch bleiben die Schlussfolgerungen bzgl. der mittleren Zeittrends der Inanspruchnahmeraten (2016–2019) nach Adjustierung nahezu unverändert. Gleiches gilt für die KV-spezifischen Abweichungen von diesen mittleren Zeittrends (Tab. B-11).

## Diskussion

Zusammenfassend ist festzustellen, dass die Inanspruchnahme von PV im Bundesgebiet von 2016 bis 2019 insgesamt weiter zunahm, womit sich der Ausbau der PV in Deutschland fortsetzte.

Es fällt auf, dass die spezialisierte PV, insbesondere deren ambulante Form SAPV, aber auch die stationäre PV dabei (weiter) deutlich zunahmen, gleichzeitig die primär hausärztlich getragene AAPV (weiter) abnahm. Inwieweit der AAPV-Rückgang durch die ab dem 4. Quartal 2017 abrechenbare BQKPmV (über-)ausgeglichen werden kann, lässt sich noch nicht abschließend beurteilen und sollte weiter untersucht werden. Die Hospizinanspruchnahme blieb gleich. Die so charakterisierte Entwicklung der PV deckt sich weitgehend mit den Beobachtungen anderer Studien, die jedoch auf einzelne Regionen und die ambulante PV konzentriert waren [7–10].

Die regionale Heterogenität/Variabilität der Inanspruchnahme von PV wurde in der Vergangenheit bereits beschrieben [1, 9, 11, 12] und ist angesichts der Zielstellung einer flächendeckenden Versorgung in ihrer Entwicklung weiter zu beobachten. Auch die regionale Variabilität einzelner PV-Formen, weniger der PV insgesamt, war 2019 zwischen den KVen weiterhin hoch. Bei SAPV und Hospizen nahm sie von 2016 auf 2019 etwas ab, während sie bei AAPV und stationärer PV zunahm. Beachtenswert sind außerdem die vom Bundestrend der PV-Inanspruchnahme abweichenden Zeittrends in einigen KVen: so der überdurchschnittlich starke Rückgang der AAPV in Brandenburg, im Saarland und in Sachsen-Anhalt und der überdurchschnittliche Anstieg von SAPV in Nordrhein und in Rheinland-Pfalz. Bei der stationären PV fallen Schleswig-Holstein, Thüringen und Berlin mit einem überdurchschnittlichen Wachstum und Mecklenburg-Vorpommern mit einer Abnahme auf. Anders als im zunehmenden Bundestrend für die Inanspruchnahme von PV insgesamt wuchs diese in Niedersachsen von 2016 bis 2019 nicht.

Die Diskussion der Ergebnisse erfolgt zunächst unter den Gesichtspunkten Bedarfsdeckung und allgemeine vs. spezialisierte PV-Inanspruchnahme, bevor auf die Gründe für die regionale Variabilität eingegangen wird.

### Bedarf an Palliativversorgung gedeckt?

Mit einem Anteil von 36,2 % lag die Inanspruchnahme von PV im letzten Lebensjahr auch 2019 noch immer deutlich unter dem für Deutschland geschätzten Bedarf von 78 % [13] entsprechend der von Murtagh et al. verwendeten Schätzmethode [14], wenn man dieser Bedarfsschätzung folgen will, die aus dokumentierten Todesursachen/Erkrankungsdiagnosen abgeleitet wird und insofern weder an einem durch Versorgende festgestellten noch an einem durch Patienten artikulierten Versorgungsbedarf orientiert ist.

Anders herum waren 2019 63,8 % der VS nicht palliativ versorgt, wobei anhand der vorliegenden Daten nicht valide quantifizierbar ist, in welchen Fällen palliativer Versorgungsbedarf bestand. Gemäß Murtagh et al. [14] hätten 2019 also 42 % der VS trotz palliativen Versorgungsbedarfs als unversorgt gegolten.

Ein anderes Bild ergibt sich, wenn man berücksichtigt, dass es bei der Versorgung älterer, gebrechlicher Patienten zu einer Überschneidung von geriatrischer und palliativer Versorgung kommen kann [15], die vor allem durch eine nicht parallele Abrechnung geriatrischer und palliativer Leistungen begründet ist und zu einer Unterschätzung der AAPV führen kann. Quantifizieren lässt sich das Unterschätzungsrisiko anhand des Anteils an VS, bei denen im letzten Lebensjahr eine (haus-)ärztliche geriatrische Komplexleistung, aber keine AAPV abgerechnet wurde (s. Onlinematerial A.4): 2019 waren dies bundesdurchschnittlich 36,7 % (mit einer Spanne von Bayern mit 28,3 % bis Mecklenburg-Vorpommern und Niedersachen mit jeweils 43,5 %, Tab. B-8). Unter deren Berücksichtigung wäre 2019 ein Anteil von eindrucksvollen 67,5 % anstatt 36,2 % an VS mit PV erreicht worden (vorn liegt dadurch mit 73,7 % Niedersachsen anstelle von Bayern; hinten liegt mit 59,5 % Bremen anstelle von Sachsen-Anhalt). Mit anderen Worten: Der ungedeckte Bedarf an PV bestünde dann bundesdurchschnittlich nur noch für 10,5 % der VS.

Ausgehend von groben Schätzungen der Deutschen Gesellschaft für Palliativmedizin [9, 16] bedürfen etwa 10 % aller Menschen mit palliativem Versorgungsbedarf spezialisierter PV. Diese Bedarfsschwelle war 2019 neben Westfalen-Lippe (PKD, 27,4 %) bereits in Brandenburg (20,4 %) und Hessen (20,1 %) deutlich überschritten – und das ohne Berücksichtigung der 3,6 % ausschließlich stationär palliativ versorgten VS.

### Trend zunehmender SAPV bei abnehmender AAPV

Das generelle Phänomen einer stetig zunehmenden SAPV bei gleichzeitig abnehmender AAPV ist in allen KVen gleichermaßen bis auf Westfalen-Lippe zu beobachten. Allein in Westfalen-Lippe ist ein gleichgerichtet zunehmender Trend sowohl für AAPV als auch SAPV/PKD-Versorgung festzumachen. Hier besteht die Besonderheit, dass allgemein palliativversorgende Ärzte, die an dem in Westfalen-Lippe geltenden Palliativversorgungsvertrag teilnehmen, über die EBM-Ziffern für AAPV hinausgehende AAPV-Ziffern abrechnen können [17]. Ein vom behandelnden (Haus‑)Arzt als palliativ eingestufter Patient wird in der Regel gleichzeitig auch beim palliativmedizinischen Konsiliardienst (PKD), der anstelle eines SAPV-Teams zum Einsatz kommt, eingeschrieben.

Infolge des abnehmenden Trends bei der AAPV erhielten 2019 bundesdurchschnittlich in etwa gleich viele VS im letzten Lebensjahr (ausschließlich) allgemeine wie (ausschließlich oder auch) spezialisierte ambulante PV. Dabei gab es zahlreiche KVen, bei denen die gegenläufige Entwicklung von (zunehmender) SAPV und (abnehmender) AAPV im Jahr 2019 einen Zustand erreichte, in dem die SAPV-Inanspruchnahmerate bereits deutlich höher lag als die Rate von VS, die ausschließlich AAPV oder ausschließlich BQKPmV („maxAAPV oder maxBQKPmV“) erhielten (Tab. B‑7, B-8): Neben Westfalen-Lippe waren dies Brandenburg, Hessen, Berlin, Hamburg und Thüringen.

Inwieweit die 2017 eingeführte BQKPmV als „AAPV+“ zu einer Trendumkehr bei der allgemeinen ambulanten PV insgesamt (AAPV und BQKPmV) führen kann, ist bislang offen. Im Gegensatz zur AAPV schließt die Abrechnung von BQKPmV die gleichzeitige Verordnung von SAPV aktuell aus [18]. Im Jahr 2019 wurden bundesweit 2,4 % der VS durch die BQKPmV zusätzlich zur AAPV allgemein ambulant palliativ versorgt. Dabei variierte die BQKPmV-Inanspruchnahme regional sehr stark und es bleibt abzuwarten, welche Dynamik sie in den nächsten Jahren weiter entfaltet (vgl. dazu auch die Ergebnisse der BQKPmV-Evaluation in Niedersachsen [10]).

Angesichts des zukünftig demografiebedingt in absoluten Zahlen wachsenden Bedarfs an PV insgesamt ist der gegenläufige Trend von zunehmender SAPV und abnehmender AAPV kritisch zu bewerten. Dies gilt insbesondere mit Blick auf die dabei eingesetzten Ressourcen, die in der SAPV deutlich höher sind als in der AAPV.

### Gründe für abnehmende AAPV bei zunehmender SAPV

Die Erklärungsansätze für die Abnahme der AAPV deuten auf einen zunehmenden Rückzug der Hausärzte aus der allgemeinen palliativen Versorgung hin, die sich vor allem mit mangelnden eigenen Ressourcen für die PV konfrontiert sehen [19–21]. Ein anderer Grund für den Rückgang an AAPV kann die in der entsprechenden Region fehlende bzw. nicht ausreichend gut ausgeprägte ergänzende allgemein palliativversorgende Infrastruktur sein [19], zu der insbesondere (Palliativ‑)Pflegedienste und ambulante Hospizdienste zählen.

Zudem kann eine hohe bzw. zunehmende SAPV-Verordnungsrate aus dem Krankenhaus heraus eine geringe(re) Beteiligung von Hausärzten an der PV bewirken, da Hausärzte danach seltener an der weiteren Versorgung (mit)beteiligt sind [22–24]. Die Versorgungsstufe der AAPV wird damit häufig übersprungen. Bei einem guten Viertel der VS, die SAPV in Anspruch nehmen, erfolgte die Erstverordnung im Krankenhaus. Krankenhäuser fungieren hier als wichtige Steuerer der weiteren PV und sollten die beteiligten Versorger, insbesondere Hausärzte, bei einer solchen Entscheidung einbeziehen [25]. Die kürzlich vorgenommene Anpassung der SAPV-Richtlinie infolge der Empfehlungen der SAVOIR-Evaluationsstudie greift diesen Sachverhalt auf [26].

Eine simple Interpretation der Zunahme von SAPV könnte lauten, dass diese allein der Bedarfsentwicklung entspreche bzw. sie eine bevorstehende SAPV-Unterversorgung aufhebe (wie z. B. durch den gestiegenen Einsatz von SAPV bei nichtonkologischen Patienten). 2 Beobachtungen deuten jedoch in eine andere Richtung:

1. Auch wenn Menschen, die SAPV in Anspruch nahmen, eine höhere Morbidität aufwiesen als AAPV-Nutzer [10, 27] – so zeigten eigene explorative Analysen, dass der Charlson-Komorbiditätsindex bei SAPV-Nutzern von 2016 (9,20) auf 2019 (8,96) abnahm. Dies spiegelt wider, dass im Zeitverlauf auch weniger morbide und damit potenziell auch weniger bedürftige Menschen mit SAPV versorgt werden.
2. Es liegen empirische Hinweise vor, dass die jeweilige Entscheidung, ob AAPV oder SAPV zum Einsatz kommt, in hohem Maße von den verfügbaren hausärztlichen AAPV-Kapazitäten abhängt bzw. dass im Bereich der AAPV bestehende Versorgungslücken durch den Einsatz von SAPV geschlossen werden [19]. Dass in solchen Fällen die Indikationsvoraussetzungen (komplexes Symptomgeschehen und besonderer Versorgungsbedarf) gemäß SAPV-Richtlinie [28] entsprechend weit ausgelegt werden (müssen), ist nachvollziehbar. Das Bundesland Brandenburg mit geringer AAPV- bei gleichzeitig hoher SAPV-Inanspruchnahmerate (2019: 12,5 % vs. 20,4 %) lässt sich als ein Beispiel für dieses Phänomen anführen. Bayern hingegen ist ein Beispiel für eine infolge höherer AAPV-Kapazitäten hohe AAPV-Rate (35,8 %) bei weniger verbreiteter SAPV (12,2 %).

Neben dem fehlenden AAPV-Angebot ist ein weiterer denkbarer Grund für den verstärkten Einsatz der SAPV eine zunehmende und nicht zwingend indikationsgerechte, allerdings Bekanntheit und Verfügbarkeit voraussetzende Patienten- und Angehörigenpräferenz für SAPV.

### Gründe für die regionale Variabilität der Inanspruchnahmeraten

Die regionale Variabilität der Inanspruchnahme der PV-Formen und deren Entwicklung erfordern KV-spezifische Interpretationen. Solche Interpretationsansätze lieferten wir bereits in unserer ersten Studie zur Inanspruchnahme von PV-Formen über das Jahr 2016 [1]. Diese sollten an anderer Stelle vertieft werden, z. B. im Rahmen von Fallstudien oder Regionalkonferenzen.

Wir liefern hiermit eine KV-übergeordnete Antwort auf die Frage nach den Gründen für die regionale Variabilität von PV: Diese ist nämlich nicht durch regional unterschiedlich verteilte, auf Versorgungsbedarf wie -zugang bezogene Patienten- bzw. Wohnkreismerkmale erklärbar. Vereinfachenden potenziellen Schlüssen wie: „in Brandenburg gibt es deshalb so viel SAPV, weil der Anteil an Krebserkrankungen und damit der SAPV-Bedarf dort höher ist als in anderen Bundesländern“, ist somit eine klare Absage zu erteilen. Die trotz Adjustierung verbleibend hohe regionale Variabilität liefert den empirischen Beleg, dass Indikationsstellungen für palliative Versorgung weniger an objektiven Bedarfskriterien orientiert sind und andere als die gemessenen Einflüsse hierfür bestimmend sein müssen. Dabei ist zuvorderst an regional verfügbare Versorgungskapazitäten (inkl. Fachpersonal) zu denken. Diese werden ihrerseits mitbestimmt von den (u. a. wirtschaftlichen) Interessen von PV-Anbietern unter den geltenden regionalspezifischen Rahmenbedingungen in Form von sozialgesetzlich gerahmten wie selektivvertraglich ausgehandelten Vertrags- und Vergütungsstrukturen sowie von historisch herausgebildeten regionalspezifischen Versorgungskulturen. Diese Zusammenhänge näher zu untersuchen, kann Aufgabe zukünftiger Studien sein.

### Limitationen

Die Stärken und Schwächen dieser Studie sind im Onlinematerial zusammengefasst.

## Schlussfolgerungen

Zunehmende SAPV (und stationäre PV), sinkende AAPV und eine hohe, nicht durch bedarfs- oder zugangsbezogene Merkmale erklärbare Variabilität zwischen KVen sprechen dafür, dass sich der regional stark variierende Einsatz der PV-Formen weniger am Bedarf als an verfügbaren Versorgungskapazitäten orientiert. Daraus sollte nicht voreilig geschlussfolgert werden, dass die Indikationskriterien für den Einsatz von SAPV verschärft werden müssen. Vielmehr ist eine Orientierung von regionalspezifischen Rahmenbedingungen für Ausgestaltung und Entscheidungen über die zum Einsatz kommende Art der PV an dadurch erreichbaren patientenorientierten Outcomes anzustreben: z. B. der Möglichkeit, zuhause zu sterben sowie unnötige Krankenhausaufenthalte und aggressive Therapien am Lebensende zu vermeiden. Diese Outcomes sollten Qualitäts- und Kostenaspekte mit Opportunitätskostenbetrachtungen (d. h. unter Berücksichtigung alternativer Verwendungsmöglichkeiten für den Ressourceneinsatz) vereinen, um darauf aufbauend angemessene Strategien für die Sicherstellung einer adäquaten PV ohne Ressourcenfehlallokation zu verfolgen. Angesichts der bevölkerungsstatistisch basierten Prognose, dass in 20 Jahren etwa zweidrittel-mal mehr Menschen jährlich sterben als heute [29], und der abnehmenden personellen Ressourcen für die Versorgung liegt darin eine essenzielle Zukunftsaufgabe.

*Weitere, ausschließlich im Onlinematerial zitierte Literaturquellen:* [30–45].

## Supplementary Information





## Footnotes

1 Für eine bessere Lesbarkeit wird nur eine sprachliche Form genutzt; hierbei sind Angehörige aller Geschlechter eingeschlossen.

2 Ländlichkeit gemessen als Anteil ländlich lebender Einwohner je Kreis gemäß INKAR (Indikatoren und Karten zur Raum- und Stadtentwicklung).

3 Für einen Vergleich mit Studien, die lediglich Verordnungen (VO) von SAPV zählen und SAPV-VO aus Krankenhaus sowie in Westfalen-Lippe in Anspruch genommene spezialisierte ambulante PV nicht berücksichtigen (siehe Radbruch et al. 2015), betragen die entsprechenden Raten 7,2 % im Jahr 2016 und 9,0 % im Jahr 2019.

4 Da eine Inanspruchnahme der BQKPmV nur in 2 der 4 betrachteten Jahre möglich war, verzichten wir auf eine inferenzstatistische Prüfung der zeitlichen Entwicklung.
