# Supplementary material for: Utilization of palliative care at the end of life in Germany: temporal trend (2016–2019) and regional variability
Source: Bundesgesundheitsblatt Gesundheitsforschung Gesundheitsschutz. 2023 Mar 10;66(4):432–42. [Article in German] doi: 10.1007/s00103-023-03683-7 (PMC10063517; doi:10.1007/s00103-023-03683-7)
Supplement: Supplementary file 1 [file 103_2023_3683_MOESM1_ESM.pdf]

Onlinematerial zum Beitrag:

Inanspruchnahme von Palliativversorgung am Lebensende in Deutschland: zeitlicher Verlauf (2016-2019) und regionale Variabilität

Bianka Ditscheid[1], Franziska Meissner[1], Cordula Gebel[2], Beata Hennig[3], Ursula Marschall[3], Winfried Meißner[2], Ulrich Wedding[2], Antje Freytag[1]

[1] Institut für Allgemeinmedizin, Universitätsklinikum Jena, Deutschland

[2] Abteilung Palliativmedizin der Klinik für Innere Medizin II, Universitätsklinikum Jena, Deutschland

[3] BARMER, Wuppertal, Deutschland

Korrespondenzadresse:
PD Dr. Antje Freytag
Institut für Allgemeinmedizin
Universitätsklinikum Jena, Friedrich-Schiller-Universität Jena
Bachstr. 18, 07743 Jena, Deutschland
Antje.Freytag@med.uni-jena.de

Inhalt

A. eMETHODEN: Ergänzungen zur Methode 4

A.1 Datenquellen 4

A.2 Ein- und Ausschlusskriterien 4

A.3 Identifizierung der Inanspruchnahme der verschiedenen Formen von Palliativversorgung .. 4

A.4 Weitere Kennzahlen mit Bezug zur Palliativversorgung 10

A.5 Datenaufbereitung 12

A.6 Statistische Analysen 13

Deskriptive Analyse 13

Mittlerer Zeittrend und regionale Abweichung vom mittleren Zeittrend 13

Variabilität 13

Adjustierung der Inanspruchnahmeraten, des mittleren Zeittrends und der regionalen Abweichung vom mittleren Zeittrend um versorgungsbedarfsbezogene Patienten- und versorgungszugangsbezogene Wohnkreismerkmale 14

A.7 Unterschiede zur Publikation Ditscheid et al. 2020 15

B. eERGEBNISSE: Ergänzungen zu den Ergebnissen 17

C. eDiskussion: Ergänzungen zur Diskussion 30

C.1 Stärken und Schwächen 30

Literatur 31

Tabellenverzeichnis

Tabelle A-1: Regional gültige Abrechnungsziffern für AAPV-Leistungen ... 5
Tabelle A-2: Regional gültige Sonderziffern für SAPV-Leistungen ... 7
Tabelle A-3: Beschreibung der weiteren Kennzahlen mit Bezug zur Palliativversorgung ... 10
Tabelle A-4: Potenziell palliativversorgungsrelevante chronische Grunderkrankungen# ... 14
Tabelle B-1: Studienpopulation je Jahr und KV: Verstorbene BARMER-Versicherte (n) und deren Anteil (%) innerhalb der Studienpopulation ... 17
Tabelle B-2: Patientenmerkmale, aggregiert über Jahre und KVen (alters- und geschlechtsstandardisiert) ... 17
Tabelle B-3: Studienpopulation vs. alle Sterbefälle je Bundesland im gesamten Zeitraum 2016-2019 18
Tabelle B-4: Patientenmerkmale (außer Grunderkrankungen) je KV, über alle Jahre hinweg (alters- und geschlechtsstandardisiert) ... 18
Tabelle B-5: Patientenmerkmale (nur Grunderkrankungen) je KV, über alle Jahre hinweg (alters- und geschlechtsstandardisiert) ... 20
Tabelle B-6: Versorgungszugangs-bezogene Wohnkreismerkmale je KV ... 21
Tabelle B-7: Inanspruchnahme der Hauptversorgungsformen im Jahr 2019 und 2016 (alters- und geschlechtsstandardisiert) ... 22
Tabelle B-8: Inanspruchnahmeraten weiterer ausgewählter Indikatoren# für die Inanspruchnahme von Palliativversorgung im Jahr 2019 und 2016 – Teil A ... 24
Tabelle B-9: Inanspruchnahmeraten weiterer ausgewählter Indikatoren# für die Inanspruchnahme von Palliativversorgung im Jahr 2019 und 2016 – Teil B ... 25
Tabelle B-10: Odds Ratios der Kovariaten, die zur Adjustierung der Daten 2019 herangezogen wurden (einfache Regression mit Inanspruchnahme von PV insgesamt als abhängiger Variable) ... 27
Tabelle B-11: Adjustierter zeitlicher Trend in den Inanspruchnahmeraten gemittelt über alle KVen sowie adjustierte KV-spezifische Abweichung vom mittleren Trend# ... 28

A. eMETHODEN: Ergänzungen zur Methode

A.1 Datenquellen

Für die Analysen haben wir pseudonymisierte Versicherten (VS)-Stammdaten (Geburtsjahr, Geschlecht, Kreiskennzeichen, Versicherungsbeginn, Versicherungsende, Todesdatum), Leistungs- und Diagnosedaten aus den Bereichen ambulante vertragsärztliche Versorgung (§§ 294, 295 SGB V), Krankenhausversorgung (§ 301 SGB V), häusliche Krankenpflege (HKP, § 302 SGB V) und stationäre Hospizleistungen (§ 39a SGB V) herangezogen.

Wir ergänzten diese Daten um ausgewählte Regionsmerkmale auf Kreisebene. So spielten wir einen Kennwert für die sozioökonomische Deprivation aller 401 deutschen Kreise im Jahr 2019 zu (German Index of Socioeconomic Deprivation, GISD [1]). Dieser Deprivationsindex fasst neun Indikatoren aus drei Dimensionen sozialer Ungleichheit (Bildung, Beruf, Einkommen) zusammen und variiert zwischen 0 und 1 (hohe Werte = hohe Deprivation). Darüber hinaus haben wir einen Kennwert aus den Indikatoren und Karten zur Raum- und Stadtentwicklung zugespielt [2]: den Anteil ländlich lebender Einwohner je Kreis, der je Kreis angibt, wie groß der Anteil der Einwohner in Gemeinden mit einer Bevölkerungsdichte unter 150 Einwohner/km² ist. Der Wert geht als relative Häufigkeit in die Analysen ein und variiert entsprechend zwischen 0 und 1. Die deskriptiven Ergebnisse wurden der besseren Lesbarkeit wegen in Prozent ausgegeben.

Zur Berechnung der im Rahmen der Alters- und Geschlechtsstandardisierung notwendigen Gewichte haben wir außerdem Sterbefallstatistiken je 5-Jahres-Altersgruppe, Geschlecht, Bundesland der Gesundheitsberichterstattung des Bundes für die Jahre 2016, 2017, 2018 und 2019 herangezogen [3].

A.2 Ein- und Ausschlusskriterien

Primäres Einschlusskriterium war ein Todesdatum in den Jahren 2016 bis 2019. Das Datenfeld ‚Todesdatum‘ wurde von der Krankenkasse zur Verfügung gestellt (aggregiertes Datenfeld aus ‚Versicherungsende‘ und ‚Austrittsgrund Tod‘). Weitere Einschlusskriterien waren ein Alter von mind. 19 Jahren zum Zeitpunkt des Todes, eine vor dem Tod mind. einjährige durchgängige Versicherung bei der datenbereitstellenden Krankenkasse sowie die Verfügbarkeit der Wohnortinformation (Kreiskennzeichen). Frühere Versicherte der Deutschen BKK, die in den Jahren 2016 bis 2018 verstorben sind, haben wir ausgeschlossen, da für diese keine vollständige Datenverfügbarkeit gewährleistet werden konnte.

A.3 Identifizierung der Inanspruchnahme der verschiedenen Formen von Palliativversorgung

Im vorliegenden Projekt haben wir auch nachlaufende Leistungsabrechnungen (Abrechnungen nach dem Tod) berücksichtigt [4, 5]. Aus diesem Grund ist eine weitere Fortschreibung der Zeitreihe über das Jahr 2019 hinaus erst zu einem späteren Zeitpunkt möglich.

Allgemeine Ambulante PalliativVersorgung (AAPV)

Die AAPV-Inanspruchnahme haben wir in erster Linie über die bundesweit gültigen Gebührenordnungspositionen (GOP) des Einheitlichen Bewertungsmaßstabs (EBM) für Ärzte[1] 03370/04370 (Palliativmedizinische Ersterhebung des Patientenstatus inkl. Behandlungsplan), 03371/04371 (Zuschlag zu der Versichertenpauschale 03000/04000 für die palliativmedizinische Betreuung des Patienten in der Arztpraxis), 03372/04372 (Zuschlag zu den Gebührenordnungspositionen 01410 oder 01413 für die palliativmedizinische Betreuung in der Häuslichkeit) und 03373/04373 (Zuschlag zu den Gebührenordnungspositionen 01411, 01412 oder 01415 für die palliativmedizinische Betreuung in der Häuslichkeit) erfasst. Diese GOP sind ausschließlich von Haus- und Kinderärzten abrechenbar. Onkologen können laut Onkologie-Vereinbarung [6] bundesweit die Palliativziffer 86518 (Palliativversorgung von Tumorpatienten; ggf. mit Zusatz I (Brandenburg), P (Westfalen-Lippe) oder Z (Berlin)) bzw. für Thüringen und Sachsen die Palliativziffer 96509 (Palliativversorgung von Tumorpatienten) abrechnen. Darüber hinaus recherchierten wir weitere, je Kassenärztliche Vereinigung (KV) gültige Ziffern für AAPV-Leistungen in öffentlich zugänglichen Quellen[2], siehe Tabelle A-1.

Tabelle A-1: Regional gültige Abrechnungsziffern für AAPV-Leistungen

KV-Region	GOP	Bezeichnung GOP
Palliativmedizinische Leistungen aus HzV-Verträgen (gemäß § 73b SGB V)		
Nordrhein, Hessen, Westfalen-Lippe, Bayern, Berlin, Sachsen, Saarland	00001	Kontaktabhängige Pauschale für die hausärztliche Betreuung von Palliativpatienten
	01490	Zuschlag für Besuche von Palliativpatienten
Nordrhein, Westfalen-Lippe	01411P	Ungeplanter eiliger Besuch (Palliativpatient)
Nordrhein, Hessen, Bayern, Saarland, Westfalen-Lippe	01417	Besuch durch eine VERAH (Palliativpatient)
Nordrhein, Westfalen-Lippe, Hessen, Bayern, Saarland	03374	Höchstwert für die GOP 03372
Hessen, Bayern, Saarland	04374	Höchstwert für die GOP 04372
Bayern	03371H	Zuschlag für die palliativmedizinische Betreuung eines HZV-Patienten in der Arztpraxis in Bayern
	03372H	Zuschlag für die palliativmedizinische Betreuung eines HZV-Patienten in der Häuslichkeit in Bayern
	03373H	Zuschlag für die dringliche palliativmedizinische Betreuung eines HZV-Patienten in der Häuslichkeit in Bayern
Bayern	3730(B/C/D/E)	Rufbereitschaft am Lebensende (genehmigungspflichtig, Nachweis über Teilnahme Kurs-Weiterbildung Palliativmedizin für Ärzte (40 Stunden))
Hessen	98984	Kennzeichnung palliativmedizinische ambulante Versorgung

[1] Für eine bessere Lesbarkeit haben wir nur eine sprachliche Form genutzt; hierbei sind Angehörige aller Geschlechter eingeschlossen.

[2] Recherche anhand der Internet-Auftritte der Kassenärztlichen Vereinigungen und der regionalen Hausärzteverbände, z.T. ergänzt durch Anschreiben und Telefonate; die BARMER stellte ebenfalls Ziffern bereit.

**KV-Region**	**GOP**	**Bezeichnung GOP**
Palliativmedizinische Leistungen aus sonstigen Selektivverträgen (gemäß § 140a SGB V u.ä.)		
Nordrhein (Palliativvertrag Ersatzkassen)	91100	Pauschale HA/FA Eingangsdiagnostik
	91101	Pauschale HA/FA zus. Aufwand Betreuung Pat./Ang. Vor Ort, Überprüfung Schmerzth. Koord., je nach Hausbes. 01410-01413, 01415
	91102	HA/FA: Koordinationspauschale, je Quartal
	91103	HA/FA: Telefonisches Konsil mit QPA und/oder amb. Pflegedienst
	91110	QPA: Bereitschaftspauschale (Rund-um-die-Uhr-Erreichbarkeit)
	91111	QPA: Pauschale für eine vom HA angeforderte fernmündliche palliativmed. Beratung, je Konsilium
	91112	QPA: Pauschale für QPA bei angef. Besuch durch HA/FA zur Vermeidung stat. KH-Beh. je 01410-01413, 01415
	01410B	Besuch eines Kranken, wegen der Erkrankung ausgeführt
	01411B	Dringender Besuch wegen der Erkrankung, unverzüglich nach Bestellung ausgeführt
	01412B	Dringender Besuch / dringende Visite auf der Belegstation wegen der Erkrankung, unverzüglich nach Bestellung ausgeführt
	01413B	Besuch eines weiteren Kranken in derselben sozialen Gemeinschaft (z. B. Familie) und/oder in beschützenden Wohnheimen bzw. Einrichtungen bzw. Pflege- oder Altenheimen mit Pflegepersonal
	01415B	Dringender Besuch eines Patienten in beschützenden Wohnheimen bzw. Einrichtungen bzw. Pflege- oder Altenheimen mit Pflegepersonal wegen der Erkrankung, noch am Tag der Bestellung ausgeführt
	90200A	Palliativmedizin Wegegebühr PKK und EKK, Wegepauschale bis 2 DKM bei Tag
	90201A	Wegegebühr PKK und EKK, Wegegeld pro DKM einfacher Besuch bei Tage
	90202A	Palliativmedizin Wegegebühr PKK und EKK, Wegegeld pro DKM dringender Besuch bei Tage
	90203A	Palliativmedizin Wegegebühr PKK und EKK, Wegepauschale bis 2 DKM bei Nacht
	90204A	Wegegebühr PKK und EKK, Wegegeld pro DKM dringender Besuch bei Nacht
Westfalen-Lippe (Palliativvertrag nach § 132d SGB V vom 01.07.2013)	91501	HA/FA: Pauschale für die Eingangsdiagnostik
	91502	HA/FA: Zuschlag für einen erforderlichen Hausbesuch mit patientenbezogenem Zeitumfang von bis zu 45 Minuten (neben GOP 01410, 01411, 01412, 01415)
	91502S	HA/FA: Zuschlag für einen erforderlichen Hausbesuch mit patientenbezogenem Zeitumfang von mehr als 45 Minuten (neben GOP 01410, 01411, 01412, 01415)
	91503	HA/FA: Patientenbezogener persönlicher Austausch des koordinierenden Haus-/Facharztes mit einem Palliativarzt des PKD

KV-Region	GOP	Bezeichnung GOP
	91504	HA/FA: Zuschlag für einen erforderlichen Hausbesuch mit patientenbezogenem Zeitumfang von bis zu 45 Minuten (neben GOP 01413)
	91504S	HA/FA: Zuschlag für einen erforderlichen Hausbesuch mit patientenbezogenem Zeitumfang von mehr als 45 Minuten (neben GOP 01413)
	91505	HA/FA: Zuschlag für die Vorhaltung einer telefonischen Erreichbarkeit und/oder einer Besuchsbereitschaft des koordinierenden Arztes
	92002	HA/FA: Hospizpatient: Zuschlag bei Besuch eines Patienten in stationärem Hospiz (neben GOP 01410, 01411, 01412)
	92003	HA/FA: Hospizpatient: Zuschlag bei Besuch weiterer Patienten in stationärem Hospiz (neben GOP 01413)

Besonders Qualifizierte und Koordinierte Palliativmedizinische Versorgung (BQKPmV)

Die BQKPmV-Inanspruchnahme identifizierten wir über die bundesweit gültigen EBM-GOP 37300 (Palliativmedizinische Ersterhebung), 37302 (Koordinationspauschale), 37305 (Palliativmedizinische Betreuung von Patienten in der Häuslichkeit), 37306 (Dringender Besuch eines Palliativpatienten in der Häuslichkeit), 37314 (Konsiliarische Erörterung durch Arzt mit der Zusatzbezeichnung Palliativmedizin), 37317 (Telefonische Erreichbarkeit und Besuchsbereitschaft in kritischen Phasen), 37318 (Telefonische Beratung von mindestens 5 Minuten Dauer) und 37320 (Fallkonferenz gemäß Vereinbarung zur Palliativversorgung (Anlage 30 zum BMV-Ä)).

Spezialisierte Ambulante Palliativversorgung (SAPV)

Für die Erfassung der SAPV-Inanspruchnahme zogen wir die bundesweit gültigen EBM-GOP für die Verordnung von SAPV 01425 (Erstverordnung) und 01426 (Folgeverordnung) heran. Des Weiteren stellte uns die BARMER eine Sondertabelle mit SAPV-Leistungsabrechnungen zur Verfügung. Diese entstammt den HKP-Daten und enthielt die SAPV-Leistungen überwiegend in Form der 10-stelligen Positionsnummer [7]. Darüber hinaus gibt es regional gültige Sonderziffern, die wir in den amb-GOP-Daten identifizierten (Tabelle A-2). Die SAPV-GOP für Berlin sind erst seit 2019 regulär in den amb-GOP-Daten verfügbar. Deshalb stellte uns die BARMER für die Jahre 2016-2018 eine Sondertabelle mit SAPV-GOP aus Berlin zur Verfügung.

Tabelle A-2: Regional gültige Sonderziffern für SAPV-Leistungen

KV	GOP	Bezeichnung GOP
Nordrhein	92150	Tel./pers. Gespräch mit Betroffenen (Patienten oder deren Angehörigen)
	92151	Tel./pers. Gespräch mit an der Versorgung teilnehmendem Arzt außerhalb PCT
	92153	Koordinationspauschale
	92154	Assessmentpauschale
	92155	Teilversorgung je Pat. und je Einsatz ärztlich sowie pflegerisch
	92157	Vollständige Versorgung je Pat. und je BHT
Berlin	99060	Beratung des behandelnden Hausarztes
	99061	Beratung des/der Versicherten u./o. dessen Angehörigen

KV	GOP	Bezeichnung GOP
	99062	Beratung der ausführenden SAPV-Pflegekraft
	99063	Koordination
	99064	Additiv unterstützende Teilversorgung
	99065	Vollständige Versorgung
	99066	Hospizwochenpauschale
	99067	HB-Zuschlag für SAPV-Assistenten (Besuch, incl. Wegegelder)
	99068	HB-Zuschlag zur SNR 99067 für SAPV-Assistenten zu "Unzeiten", Mo-Sa, 19-07 Uhr
	99069	HB-Zuschlag zur SNR 99067 für SAPV-Assistenten zu "Unzeiten", So & Feiertage
Westfalen-Lippe	91512	QPA: Pauschale für einen erforderlichen Hausbesuch mit patientenbezogenem Zeitumfang von bis zu 45 Minuten (neben GOP 01410)
	91512S	QPA: Pauschale für einen erforderlichen Hausbesuch mit patientenbezogenem Zeitumfang von mehr als 45 Minuten (neben GOP 01410)
	91512T	QPA: Pauschale für einen erforderlichen Hausbesuch mit patientenbezogenem Zeitumfang von mehr als 90 Minuten (neben GOP 01410)
	91513	QPA: Pauschale für einen erforderlichen Hausbesuch mit patientenbezogenem Zeitumfang von bis zu 45 Minuten (neben GOP 01411)
	91513S	QPA: Pauschale für einen erforderlichen Hausbesuch mit patientenbezogenem Zeitumfang von mehr als 45 Minuten (neben GOP 01411)
	91513T	QPA: Pauschale für einen erforderlichen Hausbesuch mit patientenbezogenem Zeitumfang von mehr als 90 Minuten (neben GOP 01411)
	91514	QPA: Pauschale für einen erforderlichen Hausbesuch mit patientenbezogenem Zeitumfang von bis zu 45 Minuten (neben GOP 01412)
	91514S	QPA: Pauschale für einen erforderlichen Hausbesuch mit patientenbezogenem Zeitumfang von mehr als 45 Minuten (neben GOP 01412)
	91514T	QPA: Pauschale für einen erforderlichen Hausbesuch mit patientenbezogenem Zeitumfang von mehr als 90 Minuten (neben GOP 01412)
	91515	QPA: Zuschlag für Punktionen
	91518	QPA: Pauschale für einen erforderlichen Hausbesuch mit patientenbezogenem Zeitumfang von bis zu 45 Minuten (neben GOP 01415)
	91518S	QPA: Pauschale für einen erforderlichen Hausbesuch mit patientenbezogenem Zeitumfang von mehr als 45 Minuten (neben GOP 01415)
	91518T	QPA: Pauschale für einen erforderlichen Hausbesuch mit patientenbezogenem Zeitumfang von mehr als 90 Minuten (neben GOP 01415)
	91519	QPA: Pauschale für einen erforderlichen Hausbesuch mit patientenbezogenem Zeitumfang von bis zu 45 Minuten (neben GOP 01413)

KV	GOP	Bezeichnung GOP
	91519S	QPA: Pauschale für einen erforderlichen Hausbesuch mit patientenbezogenem Zeitumfang von mehr als 45 Minuten (neben GOP 01413)
	91519T	QPA: Pauschale für einen erforderlichen Hausbesuch mit patientenbezogenem Zeitumfang von mehr als 90 Minuten (neben GOP 01413)
	92010	QPA: Hospizpatient: Durchführung palliativmedizinisches Assessment
	92012	QPA: Hospizpatient: Besuch eines Patienten in einem stationären Hospiz (neben GOP 01410)
	92013	QPA: Hospizpatient: Besuch eines Patienten in einem stationären Hospiz (neben GOP 01411)
	92014	QPA: Hospizpatient: Besuch eines Patienten in einem stationären Hospiz (neben GOP 01412)
	92015	QPA: Hospizpatient: Zuschlag für Punktionen
	92016	QPA: Hospizpatient: Besuch weiterer Patienten in einem stationären Hospiz (neben GOP 01413)
	91511	PKD: Pauschale für die Koordinierung der Versorgung
	91516	PKD: Pauschale für SAPV-Vollversorgung bis 14 Tage
	91517	PKD: Pauschale für SAPV-Vollversorgung ab 15 Tage
	91520	PKD: Durchführung eines Erstassessments
	91521	Zuschlag zur SNR 91520 für die Anfahrt bei einer Entfernung von über 25 Kilometern (einfache Strecke)
	91522	Zuschlag zur SNR 91520 für die Anfahrt bei einer Entfernung von über 50 Kilometern (einfache Strecke)
	92011	PKD: Hospizpatient: Koordinations- und Bereitschaftspauschale

Stationäre Palliativversorgung (stationäre PV)

Die stationäre PV im Krankenhaus identifizierten wir zum einen über die Operationen- und Prozedurenschlüssel (OPS) für palliative Leistungen im Krankenhaus 8-982 (Palliativmedizinische Komplexbehandlung), 8-98e (Spezialisierte stationäre palliativmedizinische Komplexbehandlung) und 8-98h (Spezialisierte palliativmedizinische Komplexbehandlung durch einen Palliativdienst) in den Krankenhaus-OPS-Daten. Zum anderen identifizierten wir Zusatzentgelte besonderer Einrichtungen in den Krankenhaus-Entgelt-Tabellen. Hierbei zogen wir sowohl bundesweit als auch auf Bundesland-Ebene im Studienzeitraum gültige, tages- oder fallbezogene, voll- und teilstationäre Entgelte für palliative Leistungen heran (recherchiert unter https://kh-entgeltschluessel.gkv-datenaustausch.de/).

Hospiz

In Anspruch genommene stationäre Hospizleistungen identifizierten wir in der entsprechenden Leistungstabelle über den „Zuschuss stationäre Hospizbehandlung“. Die Versorgung durch ambulante Hospizdienste konnte nicht berücksichtigt werden, da diese in den Krankenkassen-Routinedaten nicht auf Versichertenebene dokumentiert wird.

Palliativversorgung insgesamt

Als VS mit PV insgesamt haben wir diejenigen VS ausgewiesen, die im letzten Lebensjahr mindestens eine der o.g. palliativen Versorgungsformen (AAPV, BQKPmV, SAPV, stationäre PV, Hospiz) in Anspruch genommen haben.

A.4 Weitere Kennzahlen mit Bezug zur Palliativversorgung

Neben den o.g. palliativen Versorgungsformen haben wir weitere Kennzahlen gebildet, um die in Anspruch genommene PV näher betrachten zu können (Tabelle A-3).

Tabelle A-3: Beschreibung der weiteren Kennzahlen mit Bezug zur Palliativversorgung

Kennzahl	Erläuterung
Palliativversorgung insgesamt	
PV nur Bundesziffern	Inanspruchnahme mindestens einer der folgenden Leistungen:<br>- AAPV nach EBM,<br>- BQKPmV,<br>- SAPV-Verordnung,<br>- palliative OPS (Krankenhaus),<br>- stationäres Hospiz.<br>Versicherte, deren PV ausschließlich über Selektivverträge (AAPV, SAPV) oder Zusatzentgelte für besondere Einrichtungen (stationäre PV) abgerechnet wurde oder die ausschließlich eine SAPV-Leistung (ohne SAPV-Verordnung, aber ggf. mit SAPV-Verordnung aus dem Krankenhaus) hatten, werden hier nicht erfasst. Mit dieser Kennzahl soll eine Annäherung an den Aufgriff von palliativ versorgten Versicherten aus anderen Routinedaten-basierten Publikationen zur Inanspruchnahme von PV und somit Vergleichbarkeit mit deren Ergebnissen hergestellt werden.
PV inkl. geriatrischer Leistung	Inanspruchnahme von mindestens einer der folgenden Leistungen:<br>- AAPV<br>- BQKPmV<br>- SAPV<br>- Stationäre PV<br>- Hospiz<br>- Geriatrische Leistung (s.u.)
AAPV	
AAPV oder BQKPmV	Inanspruchnahme von AAPV- oder BQKPmV-Leistungen (s.o. AAPV, BQKPmV)). Weitere Versorgungsformen blieben unberücksichtigt.
maxAAPV oder maxBQKPmV	Inanspruchnahme von maximal AAPV- bzw. maximal BQKPmV-Leistungen, aber keine SAPV-Leistungen. Weitere Versorgungsformen blieben unberücksichtigt.
PV gemäß Onkologie-Vereinbarung	Abrechnung der Palliativziffer nach Onkologie-Vereinbarung [6] (s.o. AAPV):<br>- GOP 86518 (alle KVen außer Sachsen und Thüringen)<br>- GOP 96509 (KVen Sachsen und Thüringen)
Geriatrische Leistung, keine AAPV-Leistung	Abrechnung mindestens einer der folgenden geriatrischen Leistungskomplexe:<br>- EBM-GOP 03360 (Hausärztlich geriatrisches Basisassessment),

Kennzahl	Erläuterung
	- EBM-GOP 03362 (Hausärztlich geriatrischer Betreuungskomplex),<br>- EBM-GOP 30980 (Abklärung vor Durchführung eines weiterführenden geriatrischen Assessments durch Hausarzt),<br>- EBM-GOP 30981 (Abklärung vor Durchführung eines weiterführenden geriatrischen Assessments durch spez. Geriatrischen Vertragsarzt),<br>- EBM-GOP 30984 (Weiterführendes geriatrisches Assessment),<br>- EBM-GOP 30985 (Zuschlag zur GOP 30984),<br>- EBM-GOP 30986 (Zuschlag zur GOP 30985),<br>- EBM-GOP 30988 (Zuschlag […] für die Einleitung und Koordination von Therapiemaßnahmen nach Durchführung eines weiterführenden geriatrischen Assessments)<br>- sowie in Westfalen-Lippe zusätzlich über EBM-GOP 30980D und EBM-GOP 30981D.<br>Eine Abrechnung von AAPV-Leistungen bei den selben Versicherten wurde ausgeschlossen.
Geriatrische oder AAPV-Leistung	Abrechnung mindestens einer der oben aufgeführten geriatrischen Leistungen oder von AAPV-Leistungen (s.o. AAPV).
SAPV	
SAPV-VO	Inanspruchnahme von SAPV auf der Basis von SAPV-Verordnungen:<br>- EBM-GOP 01424 (Erstverordnung),<br>- EBM-GOP 01426 (Folgeverordnung).
SAPV-VO aus Krankenhaus	Inanspruchnahme von SAPV, deren Erstverordnung aus dem Krankenhaus erfolgte/vom Krankenhausarzt ausgestellt wurde. Diese sind nicht in GKV (Gesetzliche Krankenversicherung)-Routinedaten abgebildet. Wir haben sie indirekt identifiziert, anhand einer erstmaligen SAPV-Leistungsabrechnung (siehe SAPV) in enger zeitlicher Verbindung (bis 7 Tage) mit der Entlassung aus dem Krankenhaus bei gleichzeitigem Fehlen einer SAPV-Erstverordnung. VS aus Westfalen-Lippe haben wir von dieser Analyse ausgeschlossen, da hier eine SAPV-Verordnung nach Muster 63 nicht nötig ist, um die spezialisierte ambulante PV zu initiieren.
SAPV ohne AAPV und ohne BQKPmV	Inanspruchnahme von SAPV ohne vor-, parallel, nachgelagerte AAPV und ohne vor-/nachgelagerte BQKPmV.
Pflegerische Versorgung	
HKPpall	Inanspruchnahme palliativer HKP-Leistungen gemäß Nr. 24a der HKP-Richtlinie. Die Identifizierung erfolgte in den HKP-Leistungstabellen anhand des bundeseinheitlichen Positionsnummernverzeichnisses für HKP [8] über die Stellen 4-6. Erfasst wurden die folgenden Leistungen:<br>- 321 (Palliative Behandlungspflege),<br>- 808 (Tagespauschale "Ambulante Palliativpflege"),<br>- B24 (Symptomkontrolle bei Palliativpatientinnen und Palliativpatienten - Leistungsbeginn-Pauschale),<br>- B25 (Symptomkontrolle bei Palliativpatientinnen und Palliativpatienten - Tagespauschale 1 – Erstverordnung),<br>- B26 (Symptomkontrolle bei Palliativpatientinnen und Palliativpatienten - Tagespauschale 2 – Folgeverordnung),<br>- B27 (Symptomkontrolle bei Palliativpatientinnen und Palliativpatienten – Finalpauschale),<br>- A89 (Symptomkontrolle als Komplexleistung (24a)),

Kennzahl	Erläuterung
	- B23 (Symptomkontrolle als Tagespauschale (24a)),<br>- B89 (Einsatzpauschale AAPV - Allgemeine ambulante Palliativversorgung (Nr. 24a)),<br>- A86 (Kontinuierliche Medikamentengabe über Schmerzpumpe im Rahmen der Symptomkontrolle (24a)),<br>- A87 (Wundversorgung als Einzelfallentscheidung im Rahmen der Symptomkontrolle (24a)).
HKP ab ambulanter PV	Inanspruchnahme von HKP-Leistungen ab der ersten ambulanten Palliativleistung (AAPV, BQKPmV, SAPV).
ambulante Pflege	Inanspruchnahme mindestens einer der folgenden ambulanten Pflegeleistungen:<br>- Pflegesachleistungen (gem. §36 SGB XI)<br>- Pflegegeld (gem. §37 SGB XI)<br>- Verhinderungspflege (gem. §39 SGB XI)
sonstige	
Ambulante PV	Inanspruchnahme von AAPV oder BQKPmV oder SAPV. Weitere Versorgungsformen blieben unberücksichtigt.
Stationäre PV ohne ambulante PV	Inanspruchnahme von stationärer PV, ohne dass zuvor oder nachher ambulante PV (AAPV, BQKPmV, SAPV) zum Einsatz kam.
Tageshospiz	Die Inanspruchnahme von Tageshospizen identifizierten wir in der entsprechenden Leistungstabelle über:<br>- den „Zuschuss teilstationäre Hospizbehandlung" bzw.<br>- den „Zuschuss ambulante Hospizbehandlung".<br>Die unter https://kh-entgeltschluessel.gkv-datenaustausch.de/ recherchierten Entgelte für Leistungen palliativmedizinischer Tageskliniken konnten wir bisher in den entsprechenden Leistungstabellen nicht identifizieren.

A.5 Datenaufbereitung

Die Daten der Studienpopulation haben wir im wissenschaftlichen Datawarehouse der BARMER mittels SAS Enterprise Guide Version 8.3 (SAS 9.4, SAS Institute Inc./Cary/NC/USA) aufbereitet. Die Aufbereitung der zugespielten Regionsmerkmale sowie die im Folgenden erläuterte Berechnung des Charlson-Komorbiditätsindex und der Standardisierungsfaktoren erfolgte in R, Version 4.1.2.

Den Charlson-Komorbiditätsindex (Charlson Comorbidity Index, CCI [9]) berechneten wir mit dem R Package *comorbidity* [10], Version 0.5.3 als gewichteten CCI, nicht als einfache Summe. Grundlage für die Berechnung waren gesicherte Diagnosen aus den Versorgungsbereichen der ambulanten ärztlichen und Krankenhausleistungen sowie alle Haupt- und Nebendiagnosen aus dem Bereich Krankenhaus, jeweils im letzten Lebensjahr. Der CCI ging als metrische Größe in die Analyse ein.

Um eine Alters- und Geschlechtsstandardisierung zu realisieren, berechneten wir je Bundesland und Jahr Standardisierungsfaktoren, die in den Analysen (vgl. nächster Abschnitt) als Gewichte herangezogen wurden. Hierzu berechneten wir ausgehend von den Sterbefallstatistiken der Gesundheitsberichterstattung des Bundes für die Jahre 2016 bis 2019, wie groß der Anteil jeder soziodemografischen Subgruppe (nach Geschlecht und 5-Jahres-Altersgruppe) an allen Sterbefällen des jeweiligen Bundeslands pro Jahr ausfiel. In einem zweiten Schritt berechneten wir dieselben Anteile für die Studienpopulation. Der Quotient des jeweiligen Anteils in der Studienpopulation durch den jeweiligen Anteil in der Allgemeinbevölkerung ergibt das (noch nicht normalisierte)

Gewicht für Verstorbene aus der jeweiligen Subgruppe[3]. Im letzten Schritt wurden die Gewichte auf Ebene der KV pro Jahr normalisiert (durch Multiplikation mit dem Quotienten aus unstandardisiertem und standardisiertem N je KV und Jahr).

A.6 Statistische Analysen

Alle Analysen führten wir mit R, Version 4.1.2, durch. Analysen, die sich auf die Studienpopulation beziehen, beruhen stets auf alters- und geschlechtsstandardisierten Daten. Die berechneten Standardisierungsfaktoren bezogen wir hierfür in allen Analysen als Gewichte ein und nutzten dafür Funktionen aus dem R Package *survey* [11].

Deskriptive Analyse

Für die deskriptive Analyse der Patientenmerkmale und der Inanspruchnahme berechneten wir je nach Skalenniveau der Variablen den Anteil in % bzw. Mittelwert und Standardabweichung.

Bei der Analyse der Wohnkreismerkmale sind die Beobachtungseinheiten keine Verstorbenen, sondern Kreise. Für die beiden Kenngrößen weisen wir den kreisbevölkerungsgewichteten Mittelwert der Kreise aus. Ihre Zuordnung zu den Verstorbenen erfolgte nach deren Wohnkreis.

Mittlerer Zeittrend und regionale Abweichung vom mittleren Zeittrend

Zur Überprüfung des mittleren Zeittrends in der Inanspruchnahme der einzelnen Versorgungsformen (PV, AAPV, SAPV, stationäre PV und Hospiz) rechneten wir jeweils ein multiples Regressionsmodell (binär logistische Regression), mit der jeweiligen Zielgröße (z.B. Inanspruchnahme AAPV: ja/nein) als Outcome und den Prädiktoren KV, Jahr und der Interaktion KV*Jahr. Hierbei ging Jahr als ordinale Variable ein und KV als 17-stufige, kategoriale Variable mit Effect Coding (ungewichtetes sum coding). Bedingt durch diese Kodierung kann man am Regressionskoeffizient des Prädiktors „Jahr“ den mittleren zeitlichen Trend ablesen, also die Veränderung der jeweiligen Inanspruchnahmerate über die Zeit, gemittelt über alle 17 KVen. Die einzelnen Kontraste des Interaktionsterms erlauben eine Prüfung des Unterschieds des zeitlichen Trends jeder einzelnen KV von diesem mittleren zeitlichen Trend. Für diejenigen KVen, die eine signifikante Abweichung vom mittleren Trend zeigten, schlossen wir eine Simple Slope Analyse an. Simple Slope Analysen dienen dem tieferen Verständnis der Ergebnisse von Regressionsanalysen mit Interaktionsterm. Sie beinhalten die Berechnung des „Slopes“, d.h. des Anstiegs/Abfalls der Regressionsgerade (hier: die mit den Jahren ansteigende/abfallende Inanspruchnahme) für bestimmte Werte der moderierenden Variable (hier: bestimmte KVen). Mithilfe dieser einzelnen Simple Slopes können wir Aussagen über die (in der jeweiligen KV eben signifikant vom mittleren Trend abweichende) zeitliche Entwicklung der Inanspruchnahme treffen.

Variabilität

Wir untersuchten für alle Hauptversorgungsformen (PV, AAPV, BQKPmV, SAPV, stat. PV und Hospiz) die Variabilität der Inanspruchnahmeraten zwischen den KVen, und zwar jeweils im Jahr 2019 und 2016. Hierfür bestimmten wir zunächst den Mittelwert und die Standardabweichung der 17 KV-spezifischen Inanspruchnahmeraten je Versorgungsform und Jahr. Anschließend berechneten wir die

[3] Da Sterbefallstatistiken lediglich je Bundesland veröffentlicht werden, nicht je KV, waren diese noch nicht normalisierten Gewichte für die KVen Nordrhein und Westfalen-Lippe identisch.

Variationskoeffizienten (VK), jeweils als Quotient aus Standardabweichung geteilt durch Mittelwert [4]. Der VK ist ein relatives Streuungsmaß und erlaubt so auch einen Vergleich der Variabilität von Inanspruchnahmeraten, die sich in ihrem Mittelwert deutlich unterscheiden. So bedeutet ein Variationskoeffizient von 0,25 (25%), dass die mittlere Abweichung der 17 KV-spezifischen Inanspruchnahmeraten vom Mittelwert aller KVen genau 25%, also ein Viertel des Mittelwerts beträgt.

Tab. 2 (im Hauptmanuskript) zeigt die ermittelten VK in Prozent für das Jahr 2019, sowie die Differenz zu 2016. Ergänzend dargestellt werden die in die Berechnung des VK eingeflossenen Mittelwerte der KVen sowie, als Ergänzung, das Minimum und Maximum je KV, und der Mittelwert über alle bundesweit Verstorbenen im Jahr 2019.

Adjustierung der Inanspruchnahmeraten, des mittleren Zeittrends und der regionalen Abweichung vom mittleren Zeittrend um versorgungsbedarfsbezogene Patienten- und versorgungszugangsbezogene Wohnkreismerkmale

Wir adjustierten die Inanspruchnahmeraten im Jahr 2019 um versorgungsbedarfsbezogene Patientenmerkmale sowie versorgungszugangsbezogene Wohnkreismerkmale. Auf diese Weise überprüften wir die Hypothese, die Variation der PV-Inanspruchnahmeraten zwischen den KVen sei größtenteils durch unterschiedlich verteilte Versorgungsbedarfe begründet, auf ihre empirische Evidenz. Wir adjustierten auch die mittleren Zeittrends der Inanspruchnahmeraten und die regionalen Abweichungen vom mittleren Zeittrend.

Die verwendeten Adjustierungsgrößen umfassten in GKV (Gesetzliche Krankenversicherung)-Routinedaten verfügbare soziodemografische Merkmale und Patientencharakteristika aus dem letzten Lebensjahr sowie Merkmale des Wohnorts, von denen Einfluss auf Indikation und Umfang palliativen Versorgungsbedarfs sowie Zugang zu palliativem Versorgungsangebot ausgehen kann. Wir stellten literaturbasiert [12–14] bzw. durch Expertenmeinung gestützt folgende Liste an Adjustierungsgrößen zusammen: Alter (metrisch), Geschlecht (binär), Pflegebedarf (gemessen als dreistufige kategoriale Variable: Leben in der Häuslichkeit ohne Pflegegrad, Leben in der Häuslichkeit mit Pflegegrad bzw. Leben im Pflegeheim (Zeitpunkt: Beginn des letzten Lebensjahres; Pflegegrad: letzter dokumentierter Pflegegrad)), die aggregierte Morbidität (gemessen als gewichteter Charlson Komordiditätsindex, CCI) das Vorhandensein (dichotom) einer Krebs- und/oder einer anderen potenziell palliativversorgungsrelevanten chronischen Grunderkrankung (Tabelle A-4), sowie als Merkmale des Versorgungszugangs der sozioökonomische Deprivationsgrad (GISD) und der Anteil ländlich lebender Einwohner des Wohnkreises des Verstorbenen (beide metrisch).

Tabelle A-4: Potenziell palliativversorgungsrelevante chronische Grunderkrankungen[#]

Grunderkrankung	ICD-10-GM Codes
Tumorerkrankung	C00*-C97*
Herz-Kreislauf-Erkrankung	I0*-I6*
Herzinsuffizienz	I50*
Zerebrovaskuläre Erkrankung	I60*-I69*
Nierenerkrankung	N17*-N18*, N28*, I12*-I13*
Lebererkrankung	K70*-K77*
Atemwegserkrankung	J09*-J18*, J20*-J22*, J40*-J47*, J96*

[4] Dies impliziert, dass ein- und dieselbe Standardabweichung bei einem höheren Mittelwert einen geringeren VK ergibt. In anderen Worten: Bei einem höheren Mittelwert der KVen, also einer höheren Inanspruchnahme-rate, braucht es auch eine höhere Standardabweichung, um einen VK ähnlicher Größenordnung zu erzeugen.

**Grunderkrankung**	**ICD-10-GM Codes**
Neurodegenerative Erkrankung	G10*, G20*, G35*, G122*, G231-G233*
Demenz, Alzheimer, Senilität	F00*-F03*, G30*, R54*
HIV, AIDS	B20*-B24*

#identifiziert anhand einer mindestens einmalig kodierten Diagnose (gesichert ambulant, Krankenhaus-Haupt- oder -Nebendiagnose) im letzten Lebensjahr; die unterschiedenen Krankheitsentitäten orientierten sich an Murtagh et al. 2014 [15].

Nicht erfassbar über die uns zur Verfügung stehenden Daten sind andere relevante Prädiktoren für die Inanspruchnahme von PV wie bspw. Symptomlast und Angehörigenunterstützung.

Auch überprüften wir den Zusammenhang zwischen den ausgewählten versorgungsbedarfs-bezogenen Patienten- und versorgungszugangsbezogenen Wohnkreismerkmalen mit der Inanspruchnahme von PV (Tabelle B-10).

Zu Illustrationszwecken berechneten wir basierend auf der soeben geschilderten Liste der Adjustierungsgrößen adjustierte Inanspruchnahmeraten für das Jahr 2019. Ausgegeben wurden die *Marginal Predictive Means*, also die Inanspruchnahmeraten, die sich rechnerisch ergeben, wenn in allen KVen die gleiche (nämlich die über die gesamte Studienpopulation des Jahres 2019 hinweg beobachtete) Verteilung der Patienten- und Wohnkreismerkmale angenommen wird. Diese Methode der Berechnung (*marginal standardization*) ist in diesem Fall anderen Methoden der Bestimmung adjustierter Werte vorzuziehen [16]. Für die Berechnung nutzten wir die Funktion *svypredmeans* aus dem R Package *survey* [11].

Um den mittleren Zeittrend und die regionale Abweichung vom mittleren Zeittrend für den Einfluss von Patienten- und Wohnortcharakteristika zu kontrollieren, wurden die Adjustierungsgrößen als zusätzliche Prädiktoren in das im Abschnitt „Mittlerer Zeittrend und regionale Abweichung vom mittleren Zeittrend“ (siehe Seite 13) geschilderte multiple Regressionsmodell aufgenommen. Bei zwei der Adjustierungsgrößen kann dabei davon ausgegangen werden, dass sich der Zusammenhang mit der Inanspruchnahme über die Jahre verändert hat: Krebserkrankung und Pflegebedarf. Um dafür ebenfalls zu kontrollieren, wurde für diese, und nur für diese, nicht nur der (dann mit gewichteter Effektkodierung versehene) Haupteffekt, sondern auch die Interaktion mit Jahr als Prädiktor aufgenommen. Es wurde geprüft, ob sich die statistischen Schlussfolgerungen hinsichtlich der (regionalen Abweichung von den) mittleren Zeittrends durch Kontrolle der genannten Variablen ändern.

A.7 Unterschiede zur Publikation Ditscheid et al. 2020

Im Gegensatz zu Ditscheid et al. 2020 [17] (Ergebnisse aus SAVOIR – Teilprojekt 5) haben wir für die Datenanalyse im Projekt *pallCompare* eine mind. einjährige (in SAVOIR: zweijährige), durchgängige Versicherung bei der datenbereitstellenden Krankenkasse als Einschlusskriterium definiert und die Inanspruchnahme im gesamten letzten Lebensjahr (letzte 12 Lebensmonate) betrachtet (in SAVOIR: letzte 6 Lebensmonate). Im Bereich der ambulanten PV standen uns nun auch die SAPV-Leistungsabrechnungen aus Berlin zur Verfügung. Im Bereich der stationären PV berücksichtigten wir palliative Leistungen zusätzlich über die Abrechnungen als „Besondere Einrichtungen“.

Wir haben eine andere Methode der Standardisierung angewandt: So berechneten wir die Standardisierungsfaktoren in SAVOIR für eine bundesweite Zielpopulation, während die Zielpopulation in *pallCompare* in den KVen liegt. Entsprechend bestimmten wir die

Standardisierungsfaktoren in *pallCompare* je Bundesland und normalisierten sie auf KV-Ebene (siehe auch A.5). Mit diesem Vorgehen tragen wir der Tatsache Rechnung, dass die Abweichung der demografischen Struktur der verstorbenen BARMER-Versicherten von der demografischen Struktur der Allgemeinbevölkerung von Region zu Region unterschiedlich ausfallen kann [18].

B. eERGEBNISSE: Ergänzungen zu den Ergebnissen

Tabelle B-1: Studienpopulation je Jahr und KV: Verstorbene BARMER-Versicherte (n) und deren Anteil (%) innerhalb der Studienpopulation

	Jahr				
	**2016**	**2017**	**2018**	**2019**	**Alle Jahre**
**Kassenärztliche Vereinigung**					
Baden-Württemberg	8.105 (8,3%)	8.508 (8,5%)	8.738 (8,5%)	9.533 (8,1%)	34.884 (8,4%)
Bayern	12.035 (12,4%)	12.182 (12,2%)	12.462 (12,1%)	15.413 (13,1%)	52.092 (12,5%)
Berlin	4.444 (4,6%)	4.586 (4,6%)	4.900 (4,8%)	5.753 (4,9%)	19.683 (4,7%)
Brandenburg	3.518 (3,6%)	3.812 (3,8%)	3.890 (3,8%)	4.481 (3,8%)	15.701 (3,8%)
Bremen	428 (0,4%)	500 (0,5%)	480 (0,5%)	547 (0,5%)	1.955 (0,5%)
Hamburg	2.077 (2,1%)	2.093 (2,1%)	2.241 (2,2%)	2.267 (1,9%)	8.678 (2,1%)
Hessen	8.426 (8,7%)	8.704 (8,7%)	8.639 (8,4%)	9.906 (8,4%)	35.675 (8,5%)
Mecklenburg-Vorpommern	2.194 (2,3%)	2.230 (2,2%)	2.411 (2,3%)	2.935 (2,5%)	9.770 (2,3%)
Niedersachsen	8.923 (9,2%)	8.946 (9,0%)	9.365 (9,1%)	12.608 (10,7%)	39.842 (9,5%)
Nordrhein	15.297 (15,7%)	15.281 (15,3%)	15.866 (15,4%)	17.017 (14,5%)	63.461 (15,2%)
Rheinland-Pfalz	5.117 (5,3%)	5.300 (5,3%)	5.377 (5,2%)	5.801 (4,9%)	21.595 (5,2%)
Saarland	1.250 (1,3%)	1.273 (1,3%)	1.290 (1,3%)	1.406 (1,2%)	5.219 (1,3%)
Sachsen	4.495 (4,6%)	4.871 (4,9%)	4.989 (4,8%)	5.830 (5,0%)	20.185 (4,8%)
Sachsen-Anhalt	2.810 (2,9%)	2.860 (2,9%)	3.109 (3,0%)	3.441 (2,9%)	12.220 (2,9%)
Schleswig-Holstein	4.536 (4,7%)	4.657 (4,7%)	4.793 (4,6%)	5.067 (4,3%)	19.053 (4,6%)
Thüringen	2.470 (2,5%)	2.636 (2,6%)	2.768 (2,7%)	3.224 (2,7%)	11.098 (2,7%)
Westfalen-Lippe	11.135 (11,4%)	11.146 (11,2%)	11.806 (11,4%)	12.207 (10,4%)	46.294 (11,1%)
**Deutschland**	**97.260 (100,0%)**	**99.585 (100,0%)**	**103.124 (100,0%)**	**117.436 (100,0%)**	**417.405 (100,0%)**

Anmerkung: Aufgrund unserer Art der Standardisierung sind die unstandardisierten n je KV & Jahr exakt gleich den standardisierten n je KV & Jahr. KV: Kassenärztliche Vereinigung

Tabelle B-2: Patientenmerkmale, aggregiert über Jahre und KVen (alters- und geschlechtsstandardisiert)

	**N = 417.405**
Geschlecht (Anteil weiblich), n (%)	211.851 (50,8%)
Alter (metrisch), MW (S)	78,64 (12,8)
CCI, gewichtet, MW (S)	6,43 (4,1)
Tumor, n (%)	173.131 (41,5%)
Herz-Kreislauf-Erkrankung, n (%)	378.431 (90,7%)
Herzinsuffizienz, n (%)	188.013 (45,0%)
Zerebrovaskuläre Erkrankung, n (%)	143.913 (34,5%)
Nierenerkrankung, n (%)	209.451 (50,2%)
Lebererkrankung, n (%)	84.635 (20,3%)
Atemwegserkrankung, n (%)	256.008 (61,3%)
Neurodegenerative Erkrankung, n (%)	30.944 (7,4%)
Demenz, Alzheimer, Senilität, n (%)	186.375 (44,7%)
HIV, AIDS, n (%)	620 (0,1%)
Pflegebedürftigkeit, n (%)	
Häuslichkeit ohne Pflegegrad	117.503 (28,2%)
Häuslichkeit mit Pflegegrad	226.115 (54,2%)
Pflegeheim	73.787 (17,7%)

CCI: Charlson Comorbidity Index, KV: Kassenärztliche Vereinigung, MW: Mittelwert, S: Standardabweichung

Tabelle B-3: Studienpopulation vs. alle Sterbefälle je Bundesland im gesamten Zeitraum 2016-2019

	Studienpopulation		Verstorbene im BL laut gbe-Bund		Differenz	
**Bundesland**	**Anzahl**	**%**	**Anzahl**	**%**	**absolut**	**relativ**
Baden-Württemberg	34.884	8,4	436.064	11,7	−3,4	−28,7
Bayern	52.092	12,5	529.838	14,2	−1,8	−12,4
Berlin	19.683	4,7	138.216	3,7	1,0	26,9
Brandenburg	15.701	3,8	126.827	3,4	0,4	10,3
Bremen	1.955	0,5	31.210	0,8	−0,4	−44,2
Hamburg	8.678	2,1	70.123	1,9	0,2	10,3
Hessen	35.675	8,5	262.968	7,1	1,5	20,9
Mecklenburg-Vorpommern	9.770	2,3	84.538	2,3	0,1	3,0
Niedersachsen	39.842	9,5	374.478	10,1	−0,5	−5,2
Nordrhein-Westfalen	109.755	26,3	820.302	22,1	4,2	19,2
Rheinland-Pfalz	21.595	5,2	188.248	5,1	0,1	2,2
Saarland	5.219	1,3	53.399	1,4	−0,2	−12,9
Sachsen	20.185	4,8	218.590	5,9	−1,0	−17,7
Sachsen-Anhalt	12.220	2,9	129.606	3,5	−0,6	−16,0
Schleswig-Holstein	19.053	4,6	138.583	3,7	0,8	22,5
Thüringen	11.098	2,7	116.313	3,1	−0,5	−15,0
**Deutschland**	**417.405**	**100,0**	**3.719.303**	**100,0**		

BL: Bundesland, gbe-Bund: Gesundheitsberichterstattung des Bundes

Tabelle B-4: Patientenmerkmale (außer Grunderkrankungen) je KV, über alle Jahre hinweg (alters- und geschlechtsstandardisiert)

							Pflegebedarf					
	Geschlecht (weiblich)		Alter		CCI (gewichtet)		Häuslichkeit ohne Pflegegrad		Häuslichkeit mit Pflegegrad		Pflegeheim	
**KV**	**%**	**Rang**	**MW(S)**	**Rang**	**MW(S)**	**Rang**	**%**	**Rang**	**%**	**Rang**	**%**	**Rang**
Baden-Württemberg	50,7	8	79,32 (12,9)	1	6,17 (4,0)	13	26,9	14	54,5	10	18,7	4
Bayern	51,0	4	79,16 (12,9)	3	6,54 (4,1)	8	31,4	2	50,6	16	18,0	5
Berlin	50,4	12	77,32 (13,2)	16	6,86 (4,2)	3	31,5	1	52,9	14	15,6	14
Brandenburg	49,4	16	77,92 (12,6)	14	6,99 (4,2)	2	27,7	9	56,8	3	15,4	15
Bremen	50,6	9	78,18 (12,9)	12	5,70 (4,0)	17	26,9	13	57,3	2	15,8	13
Hamburg	51,2	3	78,34 (13,2)	11	6,04 (4,0)	15	30,2	4	50,7	15	19,1	3
Hessen	50,8	7	78,98 (12,9)	4	6,09 (4,0)	14	27,3	11	55,3	7	17,3	9
Mecklenburg-Vorpommern	48,8	17	77,29 (12,7)	17	6,82 (4,1)	5	26,2	16	58,8	1	15,0	17
Niedersachsen	50,6	10	78,67 (12,9)	7	6,36 (4,1)	11	25,3	17	54,3	11	20,4	1
Nordrhein	51,8	1	78,87 (12,6)	6	6,32 (4,0)	12	26,4	15	56,1	5	17,5	7
Rheinland-Pfalz	50,9	5	78,91 (12,7)	5	6,42 (4,1)	10	28,1	8	54,3	12	17,6	6
Saarland	51,3	2	78,39 (12,5)	9	6,58 (4,0)	7	28,6	7	56,3	4	15,1	16
Sachsen	50,8	6	79,21 (12,4)	2	6,64 (4,0)	6	27,7	10	54,9	8	17,4	8
Sachsen-Anhalt	49,9	15	77,85 (12,5)	15	6,83 (4,1)	4	28,7	6	54,7	9	16,6	12
Schleswig-Holstein	50,4	13	78,51 (12,8)	8	5,99 (4,0)	16	30,3	3	50,2	17	19,6	2

							Pflegebedarf					
	Geschlecht (weiblich)		Alter		CCI (gewichtet)		Häuslichkeit ohne Pflegegrad		Häuslichkeit mit Pflegegrad		Pflegeheim	
**KV**	**%**	**Rang**	**MW(S)**	**Rang**	**MW(S)**	**Rang**	**%**	**Rang**	**%**	**Rang**	**%**	**Rang**
Thüringen	50,5	11	78,38 (12,4)	10	7,01 (4,1)	1	26,9	12	55,9	6	17,2	10
Westfalen-Lippe	50,3	14	78,12 (13,1)	13	6,49 (4,1)	9	29,3	5	53,9	13	16,8	11
**Deutschland**	**50,8**	**-**	**78,64 (12,8)**	**-**	**6,43 (4,1)**	**-**	**28,2**	**-**	**54,2**	**-**	**17,7**	**-**

CCI: Charlson Comorbidity Index, KV: Kassenärztliche Vereinigung, MW: Mittelwert, S: Standardabweichung

Das mittlere Alter der in den Jahren 2016-2019 insgesamt Verstorbenen schwankte zwischen 77,3 Jahren in Mecklenburg-Vorpommern bis 79,3 Jahren in Baden-Württemberg. Der Anteil verstorbener Frauen war am niedrigsten in Mecklenburg-Vorpommern mit 48,8% und am höchsten in Nordrhein mit 51,8%. Der CCI schwankte zwischen 5,7 in Bremen und 7,0 in Thüringen. Der Anteil von VS, die vor ihrem Tod ohne Pflegegrad in der Häuslichkeit lebten, reichte von 25,3% in Niedersachsen bis 31,5% in Berlin. Und der Anteil von VS, die vor ihrem Tod mit Pflegegrad in der Häuslichkeit lebten, reichte von 50,3% in Schleswig-Holstein bis 58,8% in Mecklenburg-Vorpommern. Der Anteil an VS, die ein Jahr vor ihrem Tod im Pflegeheim lebten, betrug zwischen 15,0% in Mecklenburg-Vorpommern und 20,4% in Niedersachsen.

Tabelle B-5: Patientenmerkmale (nur Grunderkrankungen) je KV, über alle Jahre hinweg (alters- und geschlechtsstandardisiert)

	Tumor		Herz-Kreislauf-Erkrankung		Herz-insuffizienz		Zerebrovaskuläre Erkrankung		Nieren-erkrankung		Leber-erkrankung		Atemwegs-erkrankung		Neurode-generative Erkrankung		Demenz, Alzheimer, Senilität		HIV, AIDS	
**KV**	**%**	**Rang**	**%**	**Rang**	**%**	**Rang**	**%**	**Rang**	**%**	**Rang**	**%**	**Rang**	**%**	**Rang**	**%**	**Rang**	**%**	**Rang**	**%**	**Rang**
Baden-Württemberg	40,7	13	89,6	13	43,4	12	33,9	12	48,0	12	20,1	10	59,8	13	7,1	11	49,6	2	0,1	11
Bayern	41,1	10	90,2	12	46,7	7	35,0	8	53,2	5	23,3	1	60,8	8	7,3	8	44,2	7	0,1	8
Berlin	43,1	3	89,5	14	44,4	9	31,5	14	55,5	3	22,0	3	62,7	6	6,9	15	43,2	10	0,5	1
Brandenburg	43,3	2	92,4	4	43,9	10	34,1	11	60,5	1	20,2	9	61,0	7	7,1	9	42,6	12	0,1	15
Bremen	39,1	17	87,6	17	37,7	17	30,8	17	40,4	17	19,8	11	58,2	16	6,1	17	39,3	17	0,2	4
Hamburg	42,0	6	87,6	16	40,4	16	33,5	13	44,8	16	16,4	17	60,2	11	6,9	14	48,6	3	0,3	2
Hessen	40,4	16	90,4	10	42,9	13	31,5	15	45,9	14	18,9	13	60,0	12	7,4	7	39,7	14	0,2	5
Mecklenburg-Vorpommern	45,1	1	91,7	7	42,9	14	35,3	6	54,5	4	20,6	6	60,7	9	7,0	13	43,8	8	0,1	13
Niedersachsen	41,0	11	90,7	8	46,8	6	34,2	10	48,0	11	18,9	14	60,4	10	7,1	12	52,9	1	0,1	10
Nordrhein	41,2	9	90,7	9	43,7	11	35,4	5	48,1	10	20,5	8	63,1	4	7,8	4	46,4	4	0,2	3
Rheinland-Pfalz	40,6	15	91,8	6	47,3	4	34,6	9	48,8	9	19,8	12	64,3	2	7,8	3	43,3	9	0,2	7
Saarland	40,6	14	91,9	5	46,8	5	39,6	1	47,5	13	22,3	2	65,7	1	8,9	1	43,0	11	0,2	6
Sachsen	40,8	12	93,1	3	48,2	3	37,5	2	52,7	7	18,6	15	55,7	17	7,4	6	44,3	6	0,0	16
Sachsen-Anhalt	41,7	8	93,4	2	48,9	2	36,9	4	53,0	6	21,8	4	59,2	14	6,9	16	39,7	15	0,0	17
Schleswig-Holstein	41,9	7	88,0	15	40,4	15	31,3	16	45,5	15	16,5	16	59,2	15	7,6	5	39,7	16	0,1	9
Thüringen	42,0	5	93,7	1	50,2	1	37,3	3	58,7	2	20,9	5	62,8	5	7,1	10	45,9	5	0,1	14
Westfalen-Lippe	42,4	4	90,3	11	45,6	8	35,1	7	50,3	8	20,5	7	64,2	3	7,9	2	40,8	13	0,1	12
**Deutschland**	**41,5**	**-**	**90,7**	**-**	**45,0**	**-**	**34,5**	**-**	**50,2**	**-**	**20,3**	**-**	**61,3**	**-**	**7,4**	**-**	**44,7**	**-**	**0,1**	**-**

KV: Kassenärztliche Vereinigung

Der Blick auf die chronischen Grunderkrankungen, die die palliative Versorgungssituation ausgelöst haben könnten, zeigt die größte Spanne bei den Nierenerkrankungen mit 40,4% in Bremen und 60,5% in Brandenburg. Eine große Spanne besteht auch bei der Herzinsuffizienz zwischen 37,7% in Bremen und 50,2% in Thüringen. Der Anteil an VS mit Demenzdiagnose variiert zwischen 39,3% in Bremen und 52,9% in Niedersachsen. Atemwegserkrankungen schwanken zwischen 55,6% in Sachsen und 65,1% im Saarland; zerebrovaskuläre Erkrankungen zwischen 30,8% in Bremen und 39,6% im Saarland. Der Anteil an VS mit einer Krebserkrankung reicht von 39,1% in Bremen bis 45,1% in Mecklenburg-Vorpommern.

Tabelle B-6: Versorgungszugangs-bezogene Wohnkreismerkmale je KV

	Deprivationsindex			Anteil ländlich lebender EW		
**KV**	**MW**	**Min**	**Max**	**MW**	**Min**	**Max**
Baden-Württemberg	0,40	0,18	0,74	13,1%	0,0%	70,3%
Bayern	0,35	0,00	0,72	31,0%	0,0%	100,0%
Berlin	0,58	0,58	0,58	0,0%	0,0%	0,0%
Brandenburg	0,64	0,42	0,85	43,6%	0,0%	100,0%
Bremen	0,67	0,61	0,96	0,0%	0,0%	0,0%
Hamburg	0,32	0,32	0,32	0,0%	0,0%	0,0%
Hessen	0,43	0,09	0,75	14,7%	0,0%	100,0%
Mecklenburg-Vorpommern	0,73	0,67	0,78	56,1%	0,0%	83,5%
Niedersachsen	0,57	0,18	0,95	32,1%	0,0%	100,0%
Nordrhein	0,56	0,28	0,91	1,8%	0,0%	30,2%
Rheinland-Pfalz	0,59	0,33	0,98	27,1%	0,0%	100,0%
Saarland	0,66	0,50	0,76	4,9%	0,0%	23,5%
Sachsen	0,60	0,44	0,72	23,5%	0,0%	46,9%
Sachsen-Anhalt	0,76	0,64	0,87	47,6%	0,0%	100,0%
Schleswig-Holstein	0,61	0,38	0,90	33,1%	0,0%	76,2%
Thüringen	0,66	0,33	0,79	43,5%	0,0%	100,0%
Westfalen-Lippe	0,62	0,36	1,00	9,1%	0,0%	67,6%

EW: Einwohner, KV: Kassenärztliche Vereinigung, Max: Maximum, Min: Minimum, MW: Mittelwert

Der durchschnittliche sozioökonomische Deprivationsindex (GISD) variierte 2019 zwischen 0,32 in Hamburg und 0,76 in Sachsen-Anhalt. Der Anteil ländlich lebender Einwohner ist mit 56,1% am höchsten in Mecklenburg-Vorpommern und am niedrigsten in Nordrhein mit 1,8% der VS.

Tabelle B-7: Inanspruchnahme der Hauptversorgungsformen im Jahr 2019 und 2016 (alters- und geschlechtsstandardisiert)

		AAPV		BQKPmV		SAPV		statPV		Hospiz		PV	
	**KV**	**p (CI)**	**Rang**	**p (CI)**	**Rang**	**p (CI)**	**Rang**	**p (CI)**	**Rang**	**p (CI)**	**Rang**	**p (CI)**	**Rang**
**2019**	Baden-Württemberg	22,1 (21,2 - 22,9)	7	6,4 (5,9 - 6,9)	3	13,2 (12,5 - 13,9)	12	8,6 (8,0 - 9,2)	12	2,9 (2,6 - 3,3)	13	34,6 (33,6 - 35,6)	12
	Bayern	35,8 (35,1 - 36,6)	1	4,3 (4,0 - 4,6)	12	12,2 (11,7 - 12,8)	15	13,3 (12,7 - 13,9)	2	1,6 (1,4 - 1,8)	17	46,1 (45,3 - 46,9)	1
	Berlin	17,9 (16,9 - 18,9)	15	5,4 (4,8 - 6,0)	8	18,4 (17,4 - 19,4)	4	11,2 (10,3 - 12,0)	5	5,4 (4,8 - 6,0)	1	35,3 (34,0 - 36,5)	8
	Brandenburg	12,5 (11,5 - 13,5)	17	4,6 (4,0 - 5,2)	11	20,4 (19,2 - 21,6)	2	11,0 (10,1 - 11,9)	6	4,3 (3,8 - 4,9)	5	32,5 (31,1 - 33,9)	14
	Bremen	20,5 (17,1 - 24,0)	11	2,8 (1,3 - 4,3)	15	10,9 (8,2 - 13,5)	16	5,1 (3,1 - 7,1)	17	2,1 (0,9 - 3,2)	16	29,6 (25,7 - 33,5)	16
	Hamburg	19,8 (18,1 - 21,4)	12	5,3 (4,3 - 6,2)	9	16,6 (15,0 - 18,2)	7	11,2 (9,9 - 12,6)	4	5,2 (4,3 - 6,2)	2	33,4 (31,4 - 35,4)	13
	Hessen	20,6 (19,8 - 21,4)	10	6,6 (6,1 - 7,1)	2	20,1 (19,3 - 20,9)	3	8,1 (7,5 - 8,6)	15	4,2 (3,8 - 4,6)	6	35,6 (34,6 - 36,5)	6
	Mecklenburg-Vorpommern	22,1 (20,6 - 23,7)	6	6,1 (5,2 - 7,0)	6	15,7 (14,4 - 17,0)	9	9,5 (8,5 - 10,6)	10	4,5 (3,8 - 5,3)	3	34,9 (33,2 - 36,7)	9
	Niedersachsen	25,0 (24,2 - 25,8)	3	5,2 (4,8 - 5,5)	10	14,6 (13,9 - 15,2)	10	7,9 (7,5 - 8,4)	16	3,2 (2,9 - 3,5)	11	35,9 (35,0 - 36,7)	4
	Nordrhein	24,2 (23,5 - 24,8)	5	2,4 (2,2 - 2,7)	16	12,8 (12,3 - 13,3)	13	9,1 (8,7 - 9,6)	11	3,8 (3,5 - 4,1)	7	35,4 (34,7 - 36,1)	7
	Rheinland-Pfalz	24,6 (23,4 - 25,7)	4	6,4 (5,8 - 7,0)	4	9,8 (9,0 - 10,6)	17	10,1 (9,3 - 10,9)	9	3,1 (2,7 - 3,6)	12	35,9 (34,6 - 37,1)	5
	Saarland	19,4 (17,3 - 21,5)	13	8,3 (6,9 - 9,8)	1	16,7 (14,7 - 18,6)	6	13,0 (11,3 - 14,8)	3	4,4 (3,4 - 5,5)	4	36,9 (34,4 - 39,5)	3
	Sachsen	19,1 (18,1 - 20,1)	14	5,5 (4,9 - 6,1)	7	14,3 (13,4 - 15,3)	11	10,4 (9,6 - 11,2)	8	2,3 (1,9 - 2,7)	15	32,1 (30,9 - 33,4)	15
	Sachsen-Anhalt	13,2 (12,1 - 14,4)	16	6,2 (5,4 - 7,0)	5	12,5 (11,4 - 13,6)	14	8,1 (7,2 - 9,0)	14	3,3 (2,7 - 3,9)	10	28,9 (27,3 - 30,4)	17
	Schleswig-Holstein	21,6 (20,5 - 22,8)	9	3,4 (2,9 - 3,9)	14	15,7 (14,7 - 16,8)	8	10,5 (9,6 - 11,4)	7	2,6 (2,2 - 3,1)	14	34,9 (33,5 - 36,2)	10
	Thüringen	22,0 (20,5 - 23,4)	8	3,5 (2,9 - 4,2)	13	17,9 (16,5 - 19,2)	5	14,6 (13,3 - 15,8)	1	3,4 (2,8 - 4,0)	9	37,2 (35,5 - 38,9)	2
	Westfalen-Lippe	26,7 (25,9 - 27,5)	2	0,8 (0,6 - 0,9)	17	27,4 (26,6 - 28,2)	1	8,6 (8,1 - 9,1)	13	3,8 (3,4 - 4,1)	8	34,6 (33,8 - 35,5)	11
	**Deutschland**	**23,9 (23,7 - 24,1)**	-	**4,4 (4,3 - 4,6)**	-	**16,0 (15,8 - 16,2)**	-	**9,9 (9,7 - 10,1)**	-	**3,4 (3,3 - 3,5)**	-	**36,2 (36,0 - 36,5)**	-
**2016**	Baden-Württemberg	25,1 (24,1 - 26,1)	6	0,0 (0,0 - 0,0)	1	10,2 (9,5 - 10,9)	14	8,4 (7,8 - 9,1)	9	2,9 (2,6 - 3,3)	10	32,8 (31,8 - 33,9)	7
	Bayern	36,6 (35,7 - 37,5)	1	0,0 (0,0 - 0,0)	1	10,4 (9,9 - 11,0)	13	11,9 (11,3 - 12,5)	1	2,1 (1,9 - 2,4)	14	43,2 (42,2 - 44,1)	1
	Berlin	20,1 (18,9 - 21,3)	14	0,0 (0,0 - 0,0)	1	16,7 (15,6 - 17,8)	4	8,7 (7,8 - 9,6)	8	5,7 (5,0 - 6,4)	1	33,0 (31,6 - 34,4)	5
	Brandenburg	17,8 (16,5 - 19,1)	16	0,0 (0,0 - 0,0)	1	17,5 (16,2 - 18,7)	2	10,0 (9,0 - 10,9)	6	4,6 (3,9 - 5,3)	4	30,5 (28,9 - 32,1)	14
	Bremen	21,6 (17,6 - 25,5)	13	0,0 (0,0 - 0,0)	1	8,0 (5,3 - 10,7)	16	7,6 (5,0 - 10,3)	16	1,7 (0,4 - 3,0)	17	27,8 (23,4 - 32,2)	16
	Hamburg	22,8 (21,0 - 24,7)	12	0,0 (0,0 - 0,0)	1	14,3 (12,7 - 15,9)	5	11,1 (9,6 - 12,5)	3	5,7 (4,7 - 6,8)	2	32,6 (30,5 - 34,7)	10
	Hessen	24,8 (23,9 - 25,7)	7	0,0 (0,0 - 0,0)	1	17,1 (16,3 - 17,9)	3	7,7 (7,1 - 8,3)	14	3,7 (3,3 - 4,1)	7	34,3 (33,3 - 35,3)	3
	Mecklenburg-Vorpommern	26,0 (24,1 - 27,9)	4	0,0 (0,0 - 0,0)	1	14,3 (12,8 - 15,7)	6	10,8 (9,5 - 12,1)	4	4,9 (4,0 - 5,8)	3	34,3 (32,2 - 36,4)	4
	Niedersachsen	29,2 (28,3 - 30,2)	2	0,0 (0,0 - 0,0)	1	12,5 (11,8 - 13,3)	10	7,5 (6,9 - 8,1)	17	3,4 (3,0 - 3,8)	9	36,1 (35,1 - 37,1)	2
	Nordrhein	25,3 (24,6 - 26,0)	5	0,0 (0,0 - 0,0)	1	9,1 (8,7 - 9,6)	15	7,9 (7,5 - 8,4)	13	3,8 (3,5 - 4,1)	5	32,5 (31,8 - 33,3)	11
	Rheinland-Pfalz	26,2 (25,0 - 27,5)	3	0,0 (0,0 - 0,0)	1	6,2 (5,5 - 6,8)	17	9,6 (8,7 - 10,4)	7	2,8 (2,4 - 3,3)	11	32,8 (31,5 - 34,1)	8
	Saarland	24,3 (21,9 - 26,7)	9	0,0 (0,0 - 0,0)	1	13,7 (11,8 - 15,6)	7	10,5 (8,8 - 12,1)	5	3,6 (2,6 - 4,6)	8	32,7 (30,1 - 35,3)	9

	AAPV		BQKPmV		SAPV		statPV		Hospiz		PV	
**KV**	**p (CI)**	**Rang**	**p (CI)**	**Rang**	**p (CI)**	**Rang**	**p (CI)**	**Rang**	**p (CI)**	**Rang**	**p (CI)**	**Rang**
Sachsen	19,2 (18,0 - 20,4)	15	0,0 (0,0 - 0,0)	1	11,6 (10,6 - 12,6)	11	8,3 (7,5 - 9,2)	10	2,0 (1,6 - 2,4)	16	27,9 (26,5 - 29,2)	15
Sachsen-Anhalt	17,4 (15,9 - 18,9)	17	0,0 (0,0 - 0,0)	1	11,5 (10,3 - 12,7)	12	8,2 (7,2 - 9,2)	11	2,7 (2,1 - 3,3)	12	27,3 (25,6 - 29,0)	17
Schleswig-Holstein	23,9 (22,7 - 25,2)	10	0,0 (0,0 - 0,0)	1	13,0 (12,0 - 14,0)	9	7,7 (6,8 - 8,5)	15	2,1 (1,7 - 2,5)	15	31,6 (30,3 - 33,0)	12
Thüringen	23,2 (21,4 - 24,9)	11	0,0 (0,0 - 0,0)	1	13,4 (12,0 - 14,8)	8	11,8 (10,6 - 13,1)	2	2,6 (2,0 - 3,3)	13	32,9 (30,9 - 34,8)	6
Westfalen-Lippe	24,6 (23,8 - 25,4)	8	0,0 (0,0 - 0,0)	1	23,8 (23,0 - 24,6)	1	8,1 (7,5 - 8,6)	12	3,8 (3,4 - 4,1)	6	31,4 (30,5 - 32,3)	13
**Deutschland**	**25,8 (25,5 - 26,0)**	**-**	**0,0 (0,0 - 0,0)**	**-**	**13,3 (13,1 - 13,5)**	**-**	**8,9 (8,7 - 9,1)**	**-**	**3,4 (3,2 - 3,5)**	**-**	**33,8 (33,5 - 34,1)**	**-**

CI: Konfidenzintervall (Confidence interval), KV: Kassenärztliche Vereinigung, p: Anteil (Proportion)

Tabelle B-8: Inanspruchnahmeraten weiterer ausgewählter Indikatoren[#] für die Inanspruchnahme von Palliativversorgung im Jahr 2019 und 2016 – Teil A

		PV nur Bundesziffern		AAPV oder BQKPmV		maxAAPV oder maxBQKPmV		PV gemäß Onkologie-Vereinbarung		Geriatrische Leistung, keine AAPV-Leistung		Geriatrische oder AAPV-Leistung		PV inkl. geriatrischer Leistung		Ambulante PV	
	KV	p (CI)	Rang	p (CI)	Rang	p (CI)	Rang	p (CI)	Rang	p (CI)	Rang	p (CI)	Rang	p (CI)	Rang	p (CI)	Rang
**2019**	Baden-Württemberg	30,9 (30,0 - 31,9)	10	25,5 (24,6 - 26,3)	7	18,2 (17,4 - 19,0)	5	2,5 (2,2 - 2,9)	3	34,3 (33,3 - 35,2)	13	56,4 (55,3 - 57,4)	14	64,1 (63,1 - 65,1)	14	31,4 (30,5 - 32,4)	10
	Bayern	38,8 (38,1 - 39,6)	1	37,7 (37,0 - 38,5)	1	28,6 (27,9 - 29,4)	1	1,9 (1,6 - 2,1)	11	28,3 (27,6 - 29,0)	17	64,1 (63,3 - 64,9)	4	69,9 (69,2 - 70,7)	5	40,9 (40,1 - 41,7)	1
	Berlin	33,7 (32,5 - 35,0)	6	20,3 (19,2 - 21,3)	15	12,9 (12,0 - 13,7)	14	4,7 (4,1 - 5,3)	1	40,1 (38,9 - 41,4)	9	58,0 (56,7 - 59,3)	11	68,0 (66,7 - 69,2)	8	31,3 (30,0 - 32,5)	11
	Brandenburg	28,4 (27,0 - 29,7)	13	15,4 (14,3 - 16,4)	17	7,7 (6,9 - 8,5)	16	2,1 (1,7 - 2,5)	7	40,2 (38,7 - 41,7)	8	52,8 (51,3 - 54,3)	17	64,7 (63,3 - 66,1)	12	28,1 (26,8 - 29,4)	15
	Bremen	26,8 (23,0 - 30,6)	14	22,0 (18,5 - 25,5)	13	16,8 (13,6 - 20,0)	6	1,9 (0,7 - 3,2)	10	32,8 (28,8 - 36,7)	15	53,3 (49,0 - 57,7)	16	59,5 (55,2 - 63,8)	17	27,7 (23,9 - 31,6)	16
	Hamburg	29,8 (27,9 - 31,7)	11	22,8 (21,1 - 24,6)	12	13,0 (11,6 - 14,4)	13	4,5 (3,6 - 5,3)	2	38,9 (36,9 - 40,9)	10	58,7 (56,6 - 60,8)	10	66,8 (64,8 - 68,8)	11	29,6 (27,7 - 31,5)	13
	Hessen	31,3 (30,3 - 32,2)	9	23,6 (22,8 - 24,5)	11	12,7 (12,0 - 13,3)	15	1,2 (1,0 - 1,4)	15	38,9 (37,9 - 39,8)	11	59,5 (58,5 - 60,5)	8	67,1 (66,2 - 68,1)	9	32,7 (31,8 - 33,7)	3
	Mecklenburg-Vorpommern	33,1 (31,3 - 34,8)	7	26,0 (24,3 - 27,6)	5	16,0 (14,6 - 17,4)	7	2,1 (1,6 - 2,6)	6	43,5 (41,6 - 45,3)	2	65,6 (63,8 - 67,4)	3	71,9 (70,2 - 73,7)	3	31,7 (30,0 - 33,4)	8
	Niedersachsen	34,2 (33,3 - 35,0)	4	27,8 (27,0 - 28,6)	3	18,6 (17,9 - 19,3)	4	2,4 (2,2 - 2,7)	4	43,5 (42,7 - 44,4)	1	68,6 (67,7 - 69,4)	1	73,7 (72,9 - 74,5)	1	33,2 (32,4 - 34,0)	2
	Nordrhein	24,7 (24,1 - 25,4)	16	25,6 (24,9 - 26,2)	6	19,0 (18,4 - 19,6)	3	2,0 (1,8 - 2,2)	8	33,3 (32,6 - 34,0)	14	57,5 (56,7 - 58,3)	13	64,2 (63,4 - 64,9)	13	31,7 (31,0 - 32,5)	7
	Rheinland-Pfalz	34,0 (32,8 - 35,3)	5	27,9 (26,7 - 29,1)	2	22,1 (21,0 - 23,2)	2	1,7 (1,4 - 2,0)	13	42,8 (41,5 - 44,1)	3	67,4 (66,1 - 68,6)	2	72,8 (71,6 - 74,0)	2	31,9 (30,7 - 33,1)	6
	Saarland	35,1 (32,5 - 37,6)	2	25,1 (22,8 - 27,4)	8	15,8 (13,8 - 17,7)	8	0,4 (0,1 - 0,7)	17	41,6 (39,0 - 44,2)	5	61,0 (58,4 - 63,6)	7	69,9 (67,5 - 72,3)	6	32,4 (30,0 - 34,9)	4
	Sachsen	28,6 (27,4 - 29,8)	12	21,9 (20,8 - 23,0)	14	14,2 (13,3 - 15,1)	11	2,2 (1,9 - 2,6)	5	42,1 (40,8 - 43,4)	4	61,2 (59,9 - 62,6)	6	68,4 (67,1 - 69,7)	7	28,5 (27,3 - 29,7)	14
	Sachsen-Anhalt	26,1 (24,6 - 27,6)	15	18,1 (16,7 - 19,4)	16	13,1 (12,0 - 14,3)	12	0,7 (0,4 - 1,0)	16	41,6 (39,9 - 43,2)	6	54,8 (53,1 - 56,5)	15	63,5 (61,9 - 65,2)	15	25,6 (24,1 - 27,1)	17
	Schleswig-Holstein	32,9 (31,6 - 34,2)	8	23,9 (22,7 - 25,1)	10	15,5 (14,5 - 16,5)	9	1,3 (1,0 - 1,7)	14	37,7 (36,4 - 39,1)	12	59,4 (58,0 - 60,8)	9	66,9 (65,5 - 68,2)	10	31,2 (29,9 - 32,5)	12
	Thüringen	34,6 (32,9 - 36,3)	3	23,9 (22,4 - 25,4)	9	14,2 (13,0 - 15,5)	10	1,8 (1,3 - 2,2)	12	41,1 (39,4 - 42,9)	7	63,1 (61,4 - 64,8)	5	71,5 (69,9 - 73,1)	4	32,1 (30,4 - 33,8)	5
	Westfalen-Lippe	12,1 (11,5 - 12,7)	17	27,0 (26,2 - 27,8)	4	4,1 (3,7 - 4,5)	17	2,0 (1,7 - 2,2)	9	31,0 (30,2 - 31,8)	16	57,7 (56,8 - 58,6)	12	62,6 (61,7 - 63,5)	16	31,5 (30,7 - 32,3)	9
	**Deutschland**	**29,7 (29,4 - 29,9)**	-	**26,3 (26,0 - 26,5)**	-	**16,6 (16,3 - 16,8)**	-	**2,1 (2,0 - 2,2)**	-	**36,7 (36,4 - 36,9)**	-	**60,6 (60,3 - 60,9)**	-	**67,5 (67,2 - 67,7)**	-	**32,6 (32,3 - 32,8)**	-
**2016**	Baden-Württemberg	29,8 (28,8 - 30,8)	9	25,1 (24,1 - 26,1)	6	19,2 (18,4 - 20,1)	5	2,7 (2,3 - 3,1)	4	31,3 (30,3 - 32,3)	15	56,4 (55,3 - 57,5)	14	62,0 (60,9 - 63,1)	15	29,4 (28,4 - 30,5)	7
	Bayern	38,8 (37,9 - 39,7)	1	36,6 (35,7 - 37,5)	1	28,7 (27,9 - 29,5)	1	2,3 (2,0 - 2,6)	6	29,6 (28,7 - 30,4)	17	66,1 (65,3 - 67,0)	3	70,3 (69,4 - 71,2)	3	39,1 (38,2 - 40,0)	1
	Berlin	31,5 (30,1 - 32,9)	6	20,1 (18,9 - 21,3)	14	13,3 (12,3 - 14,3)	13	5,7 (5,0 - 6,4)	1	38,2 (36,8 - 39,6)	6	58,3 (56,8 - 59,8)	12	66,2 (64,7 - 67,7)	10	30,0 (28,6 - 31,4)	5
	Brandenburg	27,8 (26,3 - 29,3)	12	17,8 (16,5 - 19,1)	16	9,6 (8,6 - 10,6)	16	3,0 (2,4 - 3,5)	3	38,0 (36,2 - 39,7)	9	55,7 (54,0 - 57,4)	15	63,8 (62,2 - 65,4)	12	27,1 (25,6 - 28,6)	14
	Bremen	27,6 (23,2 - 32,0)	13	21,6 (17,6 - 25,5)	13	17,4 (13,7 - 21,0)	6	1,1 (0,1 - 2,1)	16	29,7 (25,4 - 34,1)	16	51,3 (46,4 - 56,2)	17	56,1 (51,2 - 60,9)	17	25,4 (21,1 - 29,6)	15
	Hamburg	29,5 (27,5 - 31,5)	11	22,8 (21,0 - 24,7)	12	14,5 (12,9 - 16,0)	12	4,2 (3,3 - 5,1)	2	36,4 (34,3 - 38,5)	12	59,2 (56,9 - 61,4)	10	65,8 (63,7 - 68,0)	11	28,7 (26,7 - 30,7)	11
	Hessen	31,7 (30,7 - 32,7)	5	24,8 (23,9 - 25,7)	7	14,7 (13,9 - 15,4)	11	1,5 (1,2 - 1,8)	12	37,6 (36,5 - 38,6)	10	62,4 (61,3 - 63,5)	5	67,9 (66,9 - 69,0)	5	31,8 (30,7 - 32,8)	3
	Mecklenburg-Vorpommern	32,8 (30,8 - 34,9)	3	26,0 (24,1 - 27,9)	4	16,7 (15,1 - 18,4)	7	2,2 (1,6 - 2,8)	7	39,5 (37,4 - 41,7)	3	65,5 (63,5 - 67,6)	4	70,2 (68,3 - 72,2)	4	31,0 (29,0 - 33,0)	4
	Niedersachsen	34,8 (33,7 - 35,8)	2	29,2 (28,3 - 30,2)	2	21,1 (20,2 - 21,9)	3	2,3 (2,0 - 2,6)	5	38,4 (37,4 - 39,5)	5	67,7 (66,7 - 68,7)	1	71,8 (70,8 - 72,8)	1	33,6 (32,6 - 34,6)	2
	Nordrhein	23,1 (22,4 - 23,7)	16	25,3 (24,6 - 26,0)	5	20,4 (19,7 - 21,0)	4	1,9 (1,6 - 2,1)	10	33,3 (32,5 - 34,0)	14	58,6 (57,8 - 59,4)	11	63,3 (62,5 - 64,1)	13	29,5 (28,8 - 30,2)	6
	Rheinland-Pfalz	31,2 (29,9 - 32,5)	8	26,2 (25,0 - 27,5)	3	22,7 (21,5 - 23,8)	2	1,8 (1,4 - 2,2)	11	40,3 (38,9 - 41,6)	2	66,5 (65,2 - 67,8)	2	70,4 (69,1 - 71,7)	2	28,8 (27,6 - 30,1)	10
	Saarland	32,0 (29,3 - 34,6)	4	24,3 (21,9 - 26,7)	9	15,5 (13,4 - 17,5)	9	0,9 (0,3 - 1,4)	17	37,4 (34,7 - 40,2)	11	61,8 (59,0 - 64,5)	7	67,2 (64,6 - 69,9)	6	29,1 (26,6 - 31,7)	9
	Sachsen	25,5 (24,2 - 26,9)	14	19,2 (18,0 - 20,4)	15	12,9 (11,9 - 14,0)	14	1,1 (0,9 - 1,4)	15	42,1 (40,6 - 43,7)	1	61,3 (59,8 - 62,9)	9	66,2 (64,7 - 67,7)	9	24,5 (23,2 - 25,8)	16
	Sachsen-Anhalt	24,5 (22,8 - 26,1)	15	17,4 (15,9 - 18,9)	17	12,3 (11,0 - 13,6)	15	1,2 (0,8 - 1,5)	14	38,0 (36,1 - 40,0)	7	55,4 (53,5 - 57,4)	16	61,8 (59,9 - 63,7)	16	23,8 (22,2 - 25,5)	17
	Schleswig-Holstein	29,5 (28,1 - 30,9)	10	23,9 (22,7 - 25,2)	10	16,3 (15,2 - 17,4)	8	1,3 (0,9 - 1,6)	13	38,0 (36,6 - 39,4)	8	61,9 (60,5 - 63,4)	6	66,7 (65,2 - 68,1)	8	29,3 (28,0 - 30,7)	8
	Thüringen	31,3 (29,4 - 33,2)	7	23,2 (21,4 - 24,9)	11	15,3 (13,7 - 16,8)	10	2,0 (1,5 - 2,5)	9	38,5 (36,4 - 40,6)	4	61,6 (59,6 - 63,7)	8	67,2 (65,2 - 69,1)	7	28,6 (26,8 - 30,5)	12
	Westfalen-Lippe	12,4 (11,8 - 13,0)	17	24,6 (23,8 - 25,4)	8	4,6 (4,2 - 5,0)	17	2,1 (1,8 - 2,4)	8	33,4 (32,5 - 34,2)	13	58,0 (57,0 - 58,9)	13	62,2 (61,3 - 63,2)	14	28,4 (27,6 - 29,3)	13
	**Deutschland**	**28,3 (28,0 - 28,6)**	-	**25,8 (25,5 - 26,0)**	-	**17,3 (17,1 - 17,5)**	-	**2,2 (2,1 - 2,3)**	-	**35,4 (35,1 - 35,7)**	-	**61,2 (60,9 - 61,5)**	-	**66,2 (65,9 - 66,5)**	-	**30,6 (30,3 - 30,9)**	-

[#]Erläuterungen zu den Indikatoren: siehe Tabelle A-3; CI: Konfidenzintervall (Confidence interval), KV: Kassenärztliche Vereinigung, p: Anteil (Proportion)

Tabelle B-9: Inanspruchnahmeraten weiterer ausgewählter Indikatoren[#] für die Inanspruchnahme von Palliativversorgung im Jahr 2019 und 2016 – Teil B

		**HKPpall**		**HKP ab ambulanter PV**		**ambulante Pflege**		**SAPV-VO**		**SAPV-VO aus Krankenhaus**		**SAPV ohne AAPV und ohne BQKPmV**		**Stationäre PV ohne ambulante PV**		**Tageshospiz**	
	**KV**	**p (CI)**	**Rang**	**p (CI)**	**Rang**	**p (CI)**	**Rang**	**p (CI)**	**Rang**	**p (CI)**	**Rang**	**p (CI)**	**Rang**	**p (CI)**	**Rang**	**p (CI)**	**Rang**
**2019**	Baden-Württemberg	0,0 (0,0 - 0,0)	7	5,7 (5,2 - 6,2)	5	57,9 (56,9 - 58,9)	8	6,4 (5,9 - 6,9)	16	3,4 (3,0 - 3,8)	11	6,0 (5,5 - 6,4)	11	3,1 (2,7 - 3,4)	12	0,0 (0,0 - 0,0)	10
	Bayern	0,0 (0,0 - 0,0)	7	6,3 (5,9 - 6,7)	4	52,7 (51,9 - 53,5)	16	7,9 (7,5 - 8,4)	12	2,7 (2,5 - 3,0)	14	3,1 (2,9 - 3,4)	17	5,2 (4,8 - 5,5)	1	0,0 (0,0 - 0,1)	5
	Berlin	0,0 (0,0 - 0,1)	5	2,6 (2,2 - 3,1)	16	52,4 (51,1 - 53,7)	17	16,1 (15,1 - 17,0)	1	1,6 (1,2 - 1,9)	16	11,0 (10,2 - 11,8)	2	4,0 (3,5 - 4,6)	5	0,0 (0,0 - 0,0)	10
	Brandenburg	0,0 (0,0 - 0,0)	6	3,8 (3,3 - 4,4)	14	55,9 (54,4 - 57,4)	11	12,7 (11,8 - 13,7)	4	6,4 (5,7 - 7,1)	1	12,7 (11,8 - 13,7)	1	4,3 (3,7 - 4,9)	4	0,0 (0,0 - 0,1)	2
	Bremen	0,0 (0,0 - 0,0)	7	4,0 (2,3 - 5,6)	11	59,7 (55,5 - 64,0)	1	6,6 (4,5 - 8,7)	15	2,8 (1,5 - 4,2)	13	5,7 (3,7 - 7,7)	13	1,9 (0,6 - 3,1)	17	0,2 (-0,2 - 0,6)	1
	Hamburg	0,0 (0,0 - 0,0)	7	2,6 (1,9 - 3,3)	17	54,7 (52,6 - 56,8)	14	10,9 (9,6 - 12,2)	9	3,8 (3,0 - 4,7)	8	6,7 (5,7 - 7,8)	8	3,8 (3,0 - 4,6)	7	0,0 (0,0 - 0,1)	4
	Hessen	0,0 (0,0 - 0,0)	7	5,5 (5,1 - 6,0)	6	58,4 (57,5 - 59,4)	4	14,2 (13,5 - 14,9)	2	5,6 (5,1 - 6,0)	3	9,1 (8,5 - 9,7)	3	2,8 (2,4 - 3,1)	15	0,0 (0,0 - 0,0)	10
	Mecklenburg-Vorpommern	0,0 (0,0 - 0,0)	7	8,3 (7,3 - 9,3)	1	59,2 (57,3 - 61,0)	2	11,5 (10,3 - 12,7)	6	3,5 (2,8 - 4,1)	10	5,7 (4,9 - 6,6)	12	2,9 (2,3 - 3,5)	14	0,0 (0,0 - 0,1)	6
	Niedersachsen	0,0 (0,0 - 0,0)	7	4,4 (4,1 - 4,8)	10	56,8 (55,9 - 57,7)	10	11,3 (10,7 - 11,9)	8	3,4 (3,1 - 3,7)	12	5,4 (5,0 - 5,8)	14	2,6 (2,3 - 2,9)	16	0,0 (0,0 - 0,0)	9
	Nordrhein	1,9 (1,7 - 2,1)	2	3,9 (3,6 - 4,2)	13	59,0 (58,3 - 59,8)	3	8,7 (8,3 - 9,2)	10	3,6 (3,3 - 3,9)	9	6,2 (5,8 - 6,5)	10	3,6 (3,3 - 3,8)	9	0,0 (0,0 - 0,1)	3
	Rheinland-Pfalz	0,0 (0,0 - 0,1)	4	4,9 (4,4 - 5,5)	8	57,0 (55,7 - 58,3)	9	6,7 (6,0 - 7,4)	14	2,7 (2,3 - 3,1)	15	4,0 (3,5 - 4,5)	16	3,9 (3,3 - 4,4)	6	0,0 (0,0 - 0,0)	10
	Saarland	0,0 (0,0 - 0,0)	7	3,9 (2,9 - 4,9)	12	58,2 (55,6 - 60,8)	6	13,1 (11,3 - 14,8)	3	4,0 (3,0 - 5,0)	7	7,4 (6,0 - 8,7)	6	4,5 (3,4 - 5,6)	3	0,0 (0,0 - 0,0)	10
	Sachsen	0,0 (0,0 - 0,0)	7	7,0 (6,4 - 7,7)	2	55,1 (53,8 - 56,5)	13	8,7 (7,9 - 9,4)	11	4,5 (3,9 - 5,0)	5	6,6 (6,0 - 7,3)	9	3,5 (3,0 - 4,0)	10	0,0 (0,0 - 0,0)	10
	Sachsen-Anhalt	0,1 (0,0 - 0,2)	3	5,5 (4,7 - 6,2)	7	55,5 (53,8 - 57,3)	12	7,0 (6,1 - 7,8)	13	4,4 (3,7 - 5,0)	6	7,5 (6,7 - 8,4)	5	3,1 (2,5 - 3,7)	11	0,0 (0,0 - 0,1)	8
	Schleswig-Holstein	0,0 (0,0 - 0,0)	7	3,7 (3,2 - 4,2)	15	54,1 (52,7 - 55,5)	15	11,3 (10,4 - 12,2)	7	4,6 (4,0 - 5,2)	4	7,3 (6,6 - 8,1)	7	3,6 (3,1 - 4,2)	8	0,0 (0,0 - 0,0)	10
	Thüringen	0,0 (0,0 - 0,0)	7	6,9 (6,0 - 7,7)	3	58,4 (56,6 - 60,2)	5	12,6 (11,5 - 13,8)	5	5,8 (4,9 - 6,6)	2	8,2 (7,2 - 9,2)	4	5,1 (4,3 - 5,9)	2	0,0 (0,0 - 0,0)	10
	Westfalen-Lippe	3,7 (3,4 - 4,1)	1	4,9 (4,5 - 5,3)	9	58,0 (57,1 - 58,9)	7	0,3 (0,2 - 0,4)	17	n.e.	-	4,5 (4,1 - 4,8)	15	3,1 (2,8 - 3,4)	13	0,0 (0,0 - 0,1)	7
	**Deutschland**	**0,7 (0,6 - 0,7)**	**-**	**5,0 (4,9 - 5,1)**	**-**	**56,5 (56,3 - 56,8)**	**-**	**9,0 (8,9 - 9,2)**	**-**	**3,8 (3,7 - 3,9)**	**-**	**6,3 (6,2 - 6,4)**	**-**	**3,6 (3,5 - 3,7)**	**-**	**0,0 (0,0 - 0,0)**	**-**
**2016**	Baden-Württemberg	0,0 (0,0 - 0,0)	6	5,6 (5,1 - 6,1)	7	51,3 (50,2 - 52,4)	9	5,3 (4,8 - 5,8)	15	2,8 (2,5 - 3,2)	12	4,3 (3,9 - 4,8)	12	3,3 (2,9 - 3,7)	7	0,0 (0,0 - 0,0)	10
	Bayern	0,0 (0,0 - 0,0)	5	6,5 (6,1 - 7,0)	3	45,6 (44,6 - 46,5)	17	6,3 (5,9 - 6,8)	11	3,0 (2,6 - 3,3)	10	2,6 (2,3 - 2,8)	17	4,0 (3,6 - 4,3)	2	0,0 (0,0 - 0,0)	7
	Berlin	0,0 (0,0 - 0,0)	6	2,9 (2,4 - 3,4)	17	46,4 (44,9 - 47,9)	15	16,2 (15,1 - 17,3)	1	0,3 (0,2 - 0,5)	16	9,9 (9,0 - 10,8)	1	3,0 (2,5 - 3,5)	11	0,1 (0,0 - 0,1)	4
	Brandenburg	0,0 (0,0 - 0,0)	6	4,6 (3,9 - 5,3)	12	53,2 (51,5 - 55,0)	3	11,8 (10,7 - 12,9)	3	5,8 (5,1 - 6,6)	1	9,3 (8,3 - 10,3)	2	3,3 (2,7 - 3,9)	6	0,0 (0,0 - 0,0)	10
	Bremen	0,0 (0,0 - 0,0)	6	5,5 (3,3 - 7,7)	8	54,6 (49,8 - 59,5)	2	6,0 (3,6 - 8,4)	12	1,9 (0,5 - 3,3)	15	3,8 (1,9 - 5,8)	14	2,4 (0,9 - 4,0)	15	0,0 (0,0 - 0,0)	10
	Hamburg	0,0 (0,0 - 0,0)	6	3,8 (3,0 - 4,7)	16	49,2 (47,0 - 51,4)	14	8,8 (7,5 - 10,1)	8	4,0 (3,1 - 4,8)	5	5,9 (4,8 - 7,0)	5	3,9 (3,0 - 4,8)	4	0,1 (-0,1 - 0,2)	3
	Hessen	0,0 (0,0 - 0,0)	3	6,1 (5,6 - 6,6)	5	53,0 (51,9 - 54,1)	4	12,2 (11,5 - 12,9)	2	4,7 (4,2 - 5,2)	2	7,0 (6,4 - 7,5)	3	2,5 (2,1 - 2,8)	14	0,0 (0,0 - 0,0)	9
	Mecklenburg-Vorpommern	0,0 (0,0 - 0,0)	6	9,1 (7,9 - 10,3)	1	58,0 (55,8 - 60,2)	1	9,3 (8,1 - 10,5)	7	4,7 (3,8 - 5,5)	3	5,0 (4,1 - 6,0)	9	3,0 (2,3 - 3,7)	10	0,0 (0,0 - 0,0)	10
	Niedersachsen	0,0 (0,0 - 0,0)	4	4,7 (4,3 - 5,2)	11	52,6 (51,5 - 53,6)	7	9,7 (9,0 - 10,3)	5	2,8 (2,4 - 3,1)	13	4,4 (4,0 - 4,9)	11	2,4 (2,1 - 2,7)	16	0,1 (0,0 - 0,1)	2
	Nordrhein	2,2 (2,0 - 2,5)	2	4,1 (3,8 - 4,4)	14	52,8 (52,0 - 53,6)	6	5,9 (5,5 - 6,2)	14	2,8 (2,6 - 3,1)	11	4,2 (3,9 - 4,5)	13	2,9 (2,7 - 3,2)	13	0,0 (0,0 - 0,1)	5
	Rheinland-Pfalz	0,0 (0,0 - 0,0)	6	4,9 (4,3 - 5,5)	9	51,5 (50,1 - 52,9)	8	3,4 (2,9 - 3,9)	16	2,3 (1,9 - 2,7)	14	2,6 (2,1 - 3,0)	16	3,9 (3,4 - 4,5)	3	0,0 (0,0 - 0,0)	8
	Saarland	0,0 (0,0 - 0,0)	6	4,0 (2,9 - 5,1)	15	53,0 (50,2 - 55,8)	5	11,0 (9,2 - 12,8)	4	3,0 (2,1 - 3,9)	9	4,8 (3,6 - 6,0)	10	3,4 (2,4 - 4,4)	5	0,0 (0,0 - 0,0)	10
	Sachsen	0,0 (0,0 - 0,0)	6	7,3 (6,5 - 8,1)	2	49,9 (48,4 - 51,5)	13	7,5 (6,7 - 8,3)	10	3,4 (2,8 - 3,9)	7	5,3 (4,7 - 6,0)	8	3,3 (2,7 - 3,8)	9	0,0 (0,0 - 0,0)	10
	Sachsen-Anhalt	0,0 (0,0 - 0,0)	6	5,8 (4,9 - 6,6)	6	51,1 (49,1 - 53,0)	11	5,9 (5,0 - 6,7)	13	3,9 (3,2 - 4,6)	6	6,4 (5,5 - 7,3)	4	3,3 (2,6 - 4,0)	8	0,1 (0,0 - 0,2)	1
	Schleswig-Holstein	0,0 (0,0 - 0,0)	6	4,5 (3,9 - 5,2)	13	46,2 (44,8 - 47,7)	16	8,8 (7,9 - 9,6)	9	4,2 (3,6 - 4,8)	4	5,4 (4,7 - 6,1)	7	2,3 (1,9 - 2,8)	17	0,0 (0,0 - 0,0)	10

	HKPpall		HKP ab ambulanter PV		ambulante Pflege		SAPV-VO		SAPV-VO aus Krankenhaus		SAPV ohne AAPV und ohne BQKPmV		Stationäre PV ohne ambulante PV		Tageshospiz	
**KV**	**p (CI)**	**Rang**	**p (CI)**	**Rang**	**p (CI)**	**Rang**	**p (CI)**	**Rang**	**p (CI)**	**Rang**	**p (CI)**	**Rang**	**p (CI)**	**Rang**	**p (CI)**	**Rang**
Thüringen	0,0 (0,0 - 0,0)	6	6,3 (5,3 - 7,3)	4	50,2 (48,1 - 52,3)	12	9,4 (8,2 - 10,6)	6	3,0 (2,4 - 3,7)	8	5,5 (4,5 - 6,4)	6	4,3 (3,5 - 5,0)	1	0,0 (0,0 - 0,0)	10
Westfalen-Lippe	3,9 (3,5 - 4,3)	1	4,8 (4,4 - 5,3)	10	51,1 (50,1 - 52,0)	10	0,2 (0,1 - 0,2)	17	n.e.	-	3,8 (3,4 - 4,1)	15	2,9 (2,6 - 3,3)	12	0,0 (0,0 - 0,0)	6
**Deutschland**	**0,8 (0,7 - 0,9)**	-	**5,2 (5,1 - 5,4)**	-	**50,7 (50,4 - 51,1)**	-	**7,2 (7,0 - 7,3)**	-	**3,2 (3,1 - 3,3)**	-	**4,8 (4,7 - 5,0)**	-	**3,1 (3,0 - 3,2)**	-	**0,0 (0,0 - 0,0)**	-

#Erläuterungen zu den Indikatoren: siehe Tabelle A-3; CI: Konfidenzintervall (Confidence interval), KV: Kassenärztliche Vereinigung, p: Anteil (Proportion)

Zieht man lediglich bundesweit geltende Abrechnungsziffern für palliative Leistungen heran, anstatt regionale und KV-spezifische Leistungs- und Sonderziffern für ambulante und stationäre palliativmedizinische Leistungen zu berücksichtigen, so betragen die entsprechenden Anteile an palliativ versorgten VS nur 28,3% im Jahr 2016 und 29,7% im Jahr 2019 (siehe PV nur Bundesziffern). Die dadurch induzierte Unterschätzung der Inanspruchnahme liegt demnach bei etwa 6 Prozentpunkten (bzw. relativ bei -18%).

Nimmt man Westfalen-Lippe aufgrund der dort abweichenden ambulanten PV-Praxis aus der Durchschnittsrechnung für den Bund heraus, so errechnet sich eine bundesdurchschnittliche SAPV-Inanspruchnahme 2016 von 12,0% und 2019 von 14,7%.

Tabelle B-10: Odds Ratios der Kovariaten, die zur Adjustierung der Daten 2019 herangezogen wurden (einfache Regression mit Inanspruchnahme von PV insgesamt als abhängiger Variable)

	**PV**	
	**OR**	**p**
Alter	1,00	ns
Geschlecht	1,20	***
CCI (gewichtet)	1,19	***
Tumor	4,41	***
Herz-Kreislauf-Erkrankung	1,00	ns
Herzinsuffizienz	0,80	***
Zerebrovaskuläre Erkrankung	0,90	***
Nierenerkrankung	0,95	***
Lebererkrankung	1,10	***
Atemwegserkrankung	0,90	***
Neurodegenerative Erkrankung	1,09	***
Demenz, Alzheimer, Senilität	1,13	***
HIV, AIDS	0,93	ns
Pflegebedarf: Häuslichkeit mit Pflegegrad	5,51	***
Pflegebedarf: Pflegeheim	4,13	***
Deprivationsindex	0,58	***
Anteil ländlich lebender EW	1,08	**

*CCI: Charlson Comorbidity Index, EW: Einwohner, ns: nicht significant, OR: Odds ratio, p: p-Wert, PV: Palliativversorgung, *** p < .001, ** p < .01*

Tabelle B-11: Adjustierter zeitlicher Trend in den Inanspruchnahmeraten gemittelt über alle KVen sowie adjustierte KV-spezifische Abweichung vom mittleren Trend#

	AAPV		SAPV		Stat. PV		Hospiz		PV	
**Mittlerer Zeittrend**	**0,92** ***		**1,06** ***		**1,06** ***		**0,90** ***		**1,02** **	
	**Zeittrend-abweichung**	**KV-spezifischer Zeittrend**	**Zeittrend-abweichung**	**KV-spezifischer Zeittrend**	**Zeittrend-abweichung**	**KV-spezifischer Zeittrend**	**Zeittrend-abweichung**	**KV-spezifischer Zeittrend**	**Zeittrend-abweichung**	**KV-spezifischer Zeittrend**
Baden-Württemberg	1,00 ns		1,04 *	1,10 ***	0,97 +		1,00 ns		0,99 ns	
Bayern	1,03 **	0,94 ***	0,98 ns		1,03 *	1,09 ***	0,90 ***	0,81 ***	0,99 ns	
Berlin	1,02 ns		0,94 ***	1,00 ns	1,07 **	1,14 ***	0,96 ns		1,00 ns	
Brandenburg	0,93 **	0,86 ***	1,02 ns		1,03 ns		1,01 ns		1,02 ns	
Bremen	1,04 ns		1,02 ns		0,84 *	0,88 ns	0,97 ns		1,01 ns	
Hamburg	0,99 ns		0,99 ns		1,01 ns		0,98 ns		0,97 ns	
Hessen	0,96 **	0,88 ***	0,98 ns		0,99 ns		1,03 ns		0,98 *	0,99 ns
Mecklenburg-Vorpommern	1,01 ns		1,00 ns		0,93 *	0,98 ns	0,98 ns		1,00 ns	
Niedersachsen	0,97 *	0,89 ***	0,97 *	1,03 *	0,99 ns		0,95 +		0,95 ***	0,96 ***
Nordrhein	1,03 **	0,95 ***	1,04 **	1,11 ***	1,02 ns		0,97 ns		1,00 ns	
Rheinland-Pfalz	1,01 ns		1,10 ***	1,17 ***	0,97 ns		0,98 ns		1,00 ns	
Saarland	0,93 *	0,85 ***	1,00 ns		1,06 ns		1,03 ns		1,04 ns	
Sachsen	1,05 *	0,96 *	0,99 ns		1,04 +		1,06 ns		1,02 ns	
Sachsen-Anhalt	0,94 *	0,86 ***	0,96 ns		0,97 ns		1,10 +		1,00 ns	
Schleswig-Holstein	0,99 ns		0,97 ns		1,08 **	1,14 ***	1,03 ns		0,99 ns	
Thüringen	1,02 ns		1,04 ns		1,06 *	1,12 ***	1,09 ns		1,03 ns	
Westfalen-Lippe	1,11 ***	1,02 *	0,98 ns		0,98 ns		1,00 ns		1,02 +	

*#Anmerkung: Dargestellt sind Odds Ratios des mittleren Zeittrends und der Abweichung der 17 KVen vom mittleren Zeittrend mit ihrer jeweiligen Signifikanz. Für KVen, deren Zeittrend signifikant (mit p < .05) vom mittleren Zeittrend abweicht, ist zusätzlich auch der KV-spezifische Zeittrend ausgewiesen. *** p < .001, ** p < .01, * p < .05, + p < .10. KV: Kassenärztliche Vereinigung*

Nach Adjustierung ändern sich die Schlussfolgerungen bzgl. der mittleren Zeittrends nicht, mit einer einzigen Ausnahme: Der mittlere Zeittrend ist bei der Hospizinanspruchnahme nun signifikant negativ (vorher: nicht signifikant).

Bei den KV-spezifischen Abweichungen vom mittleren Zeittrends gibt es einige wenige Änderungen: AAPV: Nun weicht auch Niedersachsen ab, der Zeittrend ist hier stärker abfallend als der mittlere Trend; Sachsen weicht noch immer signifikant ab, der Trend in Sachsen ist nun aber nicht mehr ns, sondern abfallend (aber weniger stark als im Mittel der KVen). SAPV: auch in Baden-Württemberg zeigt sich nun eine stärkere Zunahme über die Zeit als im Mittel der KVen; auch bei Niedersachsen gibt es nun eine Abweichung: Hier ist die Zunahme über die Zeit weniger stark als im Mittel der KVen, aber immer noch signifikant. Stationäre PV: Bayern und Berlin zeigen nun einen stärkeren Anstieg über die Zeit, Bremen einen schwächeren (der dann auch nicht mehr signifikant ist). Hospiz: keine anderen Schlussfolgerungen bzgl. der KV-spezifischen Effekte. PV gesamt: in Hessen zeigt sich ein schwächerer Zeittrend (genauer: kein Anstieg über die Zeit); in Niedersachsen ist der abweichende Zeittrend nun nicht mehr wie vorher ns sondern sogar signifikant abfallend.

C. eDiskussion: Ergänzungen zur Diskussion

C.1 Stärken und Schwächen

Die Stärken der vorliegenden Studie liegen sowohl in der bundesweiten Betrachtung der Inanspruchnahme von PV und dem Vergleich aller 17 KVen, als auch in der umfassenden Berücksichtigung verfügbarer Abrechnungsziffern für Verordnungen und Leistungen palliativmedizinischer und -pflegerischer Versorgung. Ohne diese wird die Inanspruchnahme von PV in Deutschland massiv unterschätzt, wie es leider in diversen Studien der Fall war und weiter ist [19–23]. Darüber hinaus bringt die Differenzierung der fünf Hauptformen der PV z.B. nach der SAPV-Erstverordnung im Krankenhaus, der Abrechnung geriatrischer Leistungen, häuslicher Krankenpflegeleistungen sowie durch kombinierte Inanspruchnahme-Abfragen wie „SAPV ohne AAPV/BQKPmV“ – eine bislang nicht dagewesene Transparenz in die Inanspruchnahme palliativer Versorgungsleistungen in Deutschland.

Mit den um ausgewählte, in GKV-Routinedaten verfügbare Patienten- und Wohnkreismerkmale adjustierten regionalen Inanspruchnahmeraten wird erstmals empirisch gezeigt, dass diese versorgungsbedarfs- und versorgungszugangsbezogenen Merkmale die großen regionalen Unterschiede in der Inanspruchnahme der verschiedenen PV-Formen nicht erklären. Dies ist eine Beobachtung, aus der sich wichtige Schlüsse für die Weiterentwicklung der Palliativversorgung ziehen lassen.

Limitierend ist, dass anhand der uns vorliegenden Daten keine valide Aussage darüber möglich ist, ob palliative Versorgung überhaupt bzw. die abgerechnete Form der Palliativversorgung indiziert war bzw. objektiver Bedarf dafür bestand. Diese Einschränkung gilt grundsätzlich sowohl für palliativ versorgte wie nicht palliativ versorgte VS. Limitierend ist ebenfalls, dass die Angabe, dass PV abgerechnet wurde, keine Aussage darüber ermöglicht, inwieweit die betreffende Form der Palliativversorgung ausreichend früh und intensiv zum Einsatz kam. Entsprechend unsicher sind abgeleitete Aussagen über potenziell ungedeckten Versorgungsbedarf. Limitierend ist weiterhin, dass die Studienergebnisse trotz der vorgenommenen Alters- und Geschlechtsstandardisierung nur eingeschränkt auf die gesamte GKV-Population übertragbar sind; dies ist auf die unterschiedlichen Anteile der BARMER-Versicherten in den einzelnen KV-Regionen (vgl. Tabelle B-3: Studienpopulation vs. alle Sterbefälle je Bundesland im gesamten Zeitraum 2016-2019) und die potenziellen Spezifika der durch die BARMER geschlossenen Selektivverträge zur PV zurückzuführen. Die Zuordnung palliativer Leistungen erfolgte anhand der KV-Region der Versicherten unabhängig davon, wo die Leistung erbracht wurde.

Wir bewerteten die Daten bisher nur bis einschließlich 2019. Potenzielle Verschiebungen in der Inanspruchnahme von PV durch die SARS-CoV-2-Pandemie sind erst analysierbar, wenn 2020er und 2021er Daten verfügbar und ausgewertet sind. Erste Ergebnisse (Pallpan) deuten darauf hin, dass zwar die stationäre PV weniger in Anspruch genommen wurde, die PV insgesamt aber nicht abgenommen hat, weil das ambulante Angebot, insbesondere die SAPV stärker in Anspruch genommen wurde. Vermutlich auch wegen einer geringeren Inanspruchnahme hausärztlicher AAPV, weil diese durch die Pandemie und Digitalisierungsanforderungen stark anderweitig gebunden waren.

Literatur

1. Michalski N, Reis M, Tetzlaff F, Nowossadeck E, Hoebel J (2022) German Index of Socioeconomic Deprivation (GISD). Daten für die Jahre 1998-2019. GISD Version v2022.0.2. https://github.com/GISD-FG28/GISD/blob/master/Revisions/2022/Bund/Kreis/Kreis.csv
2. Bundesinstitut für Bau-, Stadt- und Raumforschung (2022) INKAR online – Indikatoren und Karten zur Raum- und Stadtentwicklung. https://www.inkar.de/. Zugegriffen: 30. August 2022
3. Sterbefälle, Sterbeziffern Primärquelle: Statistisches Bundesamt, Todesursachenstatistik, Fortschreibung des Bevölkerungsstandes. https://www.gbe-bund.de (Recherche nach Themen: Gesundheitliche Lage - Sterblichkeit - Mortalität und Todesursachen)
4. Ditscheid B, Storch J, Krause M, Meyer I, Freytag A (2020) Leistungs- und Abrechnungsdatum in GKV-Routinedaten: Umgang mit zeitlichen Abweichungen. Gesundheitswesen 82:20–28. https://doi.org/10.1055/a-1030-4223
5. Kremeike K, Bausewein C, Freytag A et al. (2022) DNVF-Memorandum Versorgungsforschung im letzten Lebensjahr (DNVF Memorandum: Health Services Research in the Last Year of Life). Gesundheitswesen. https://doi.org/10.1055/a-1889-4705
6. GKV-Spitzenverband, Kassenärztliche Bundesvereinigung (2009) Vereinbarung über die qualifizierte ambulante Versorgung krebskranker Patienten „Onkologie-Vereinbarung“ (Anlage 7 zum Bundesmantelvertrag-Ärzte)
7. GKV-Spitzenverband Bundeseinheitliches Positionsnummernverzeichnis für Leistungen der spezialisierten ambulanten Palliativversorgung. (Stand: 30.06.2020)
8. GKV-Spitzenverband Bundeseinheitliches Positionsnummernverzeichnis für Leistungen der häuslichen Krankenpflege und Haushaltshilfe. (Stand 28.09.2021)
9. Charlson ME, Pompei P, Ales KL, MacKenzie CR (1987) A new method of classifying prognostic comorbidity in longitudinal studies: development and validation. J Chronic Dis 40:373–383. https://doi.org/10.1016/0021-9681(87)90171-8
10. Gasparini A (2018) comorbidity: An R package for computing comorbidity scores. JOSS 3:648. https://doi.org/10.21105/joss.00648
11. Lumley T (2021) Survey. Analysis of Complex Survey Samples. R Package Version 4.1-1
12. Krause M, Ditscheid B, Lehmann T et al. (2021) Effectiveness of two types of palliative home care in cancer and non-cancer patients: A retrospective population-based study using claims data. Palliative medicine 35:1158–1169. https://doi.org/10.1177/02692163211013666
13. Just J, Schmitz M-T, Grabenhorst U, Joist T, Horn K, Weckbecker K (2022) Specialized outpatient palliative care—clinical course and predictors for living at home until death. Dtsch Arztebl Int:327–332. https://doi.org/10.3238/arztebl.m2022.0172
14. Rusche H, Kreimendahl F, Huenges B, Becka D, Rychlik R (2016) Medizinische Versorgung und Kosten im letzten Lebensjahr (Medical care and costs in the last year of life – propensity score matching of AAPV and SAPV insurants). Dtsch med Wochenschr 141:e203-e212. https://doi.org/10.1055/s-0042-114481
15. Murtagh FE, Bausewein C, Verne J, Groeneveld EI, Kaloki YE, Higginson IJ (2014) How many people need palliative care? A study developing and comparing methods for population-based estimates. Palliative medicine 28:49–58. https://doi.org/10.1177/0269216313489367
16. Muller CJ, MacLehose RF (2014) Estimating predicted probabilities from logistic regression: different methods correspond to different target populations. Int J Epidemiol 43:962–970. https://doi.org/10.1093/ije/dyu029

17. Ditscheid B, Krause M, Lehmann T et al. (2020) Palliativversorgung am Lebensende in Deutschland : Inanspruchnahme und regionale Verteilung (Palliative care at the end of life in Germany : Utilization and regional distribution). Bundesgesundheitsblatt - Gesundheitsforschung - Gesundheitsschutz 63:1502–1510. https://doi.org/10.1007/s00103-020-03240-6
18. Meissner F, Ditscheid B, & Freytag A (2022) Alters- und Geschlechtsstandardisierung bei kleinräumigen GKV-Routinedatenanalysen von verstorbenen Versicherten. Vortrag auf dem virtuellen AGENS Methodenworkshop. Universitätsklinikum Frankfurt a.M., Deutschland.
19. Radbruch L, Andersohn F, Walker J (2015) Palliativversorgung - Modul 3. Überversorgung kurativ – Unterversorgung palliativ? Analyse ausgewählter Behandlungen am Lebensende. Faktencheck Gesundheit
20. Hemmerling M, Stahmeyer JT, Schütte S, van Baal K, Stiel S (2022) Besonders qualifizierte und koordinierte palliativmedizinische Versorgung. Patientenklientel und Effekte auf die ambulante Palliativversorgung. Z Allg Med 98:337-344. https://doi.org/10.53180/zfa.2022.0337-0344
21. Gothe H, Brinkmann C, Schmedt N, Walker J, Ohlmeier C (2022) Is there an unmet medical need for palliative care services in Germany? Incidence, prevalence, and 1-year all-cause mortality of palliative care sensitive conditions: real-world evidence based on German claims data. J Public Health (Berl.) 30:711–720. https://doi.org/10.1007/s10389-020-01319-7
22. van Baal K, Stiel S, Hemmerling M et al. (2021) Ambulante Palliativversorgung in Niedersachsen – regionale Unterschiede in der Versorgung von Menschen am Lebensende anhand von Qualitätsindikatoren. Versorgungsatlas-Bericht, Nr. 21/04., Berlin
23. van Baal K, Schrader S, Schneider N et al. (2020) Quality indicators for the evaluation of end-of-life care in Germany - a retrospective cross-sectional analysis of statutory health insurance data. BMC Palliative Care 19:187. https://doi.org/10.1186/s12904-020-00679-x
